# Fine-scale variation in projected climate change presents opportunities for biodiversity conservation in Europe

**DOI:** 10.1038/s41598-021-96717-6

**Published:** 2021-08-26

**Authors:** Tomáš Hlásny, Martin Mokroš, Laura Dobor, Katarína Merganičová, Martin Lukac

**Affiliations:** 1 Faculty of Forestry and Wood Sciences, Czech University of Life Sciences Prague, Kamýcká 129, 165 00 Prague, Czechia; 2 School of Agriculture, Policy and Development, University of Reading, Reading, RG6 6AR UK

**Keywords:** Climate sciences, Environmental sciences

## Abstract

Climate change is a major threat to global biodiversity, although projected changes show remarkable geographical and temporal variability. Understanding this variability allows for the identification of regions where the present-day conservation objectives may be at risk or where opportunities for biodiversity conservation emerge. We use a multi-model ensemble of regional climate models to identify areas with significantly high and low climate stability persistent throughout the twenty-first century in Europe. We then confront our predictions with the land coverage of three prominent biodiversity conservation initiatives at two scales. The continental-scale assessment shows that areas with the least stable future climate in Europe are likely to occur at low and high latitudes, with the Iberian Peninsula and the Boreal zones identified as prominent areas of low climatic stability. A follow-up regional scale investigation shows that robust climatic refugia exist even within the highly exposed southern and northern macro-regions. About 23–31% of assessed biodiversity conservation sites in Europe coincide with areas of high future climate stability, we contend that these sites should be prioritised in the formulation of future conservation priorities as the stability of future climate is one of the key factors determining their conservation prospects. Although such focus on climate refugia cannot halt the ongoing biodiversity loss, along with measures such as resilience-based stewardship, it may improve the effectiveness of biodiversity conservation under climate change.

## Introduction

Rapid climate change induced by human activity is already changing the distribution of a great number of species^1,2^. Current commitments under the Paris COP21 agreement are unlikely to keep planetary warming below 2 °C considered safe, plus several feedback mechanisms and climate tipping points threaten to exacerbate it further^3^. Climate change is thus one of the key threats to biodiversity at all levels; from genes to biomes^4^. On the one hand, effects of climate change range from local extinctions of species with narrow ecological niches, to profound ecosystem change at the trailing edge of the shifting climatic envelope^5^. On the other hand, we already see increased species richness in areas acting as refugia and effects such as the Northern Biodiversity Paradox^6^. Recent observations from Europe show a significant decrease in mountain species richness in the Mediterranean and a concurrent increase in the boreal–temperate zone^7^. Climatic processes driving these changes include the emergence of novel climates, rapid displacement of climatic isoclines, or the divergence of temperature and precipitation vectors^8^, all resulting in significant shifts of species distribution.

Species extinctions and migration are likely to generate novel communities and thus challenge the contemporary conservation planning framework^9^, which relies on a fixed system of protected areas and conservation goals. Rapid climate change highlights the need for accommodating transient ecosystem dynamics into conservation planning^9,10^, yet practical implementation of climate change adaptation into conservation management is still rare^11^. Recent climate-adapted conservation concepts include an evaluation of species vulnerability by empirical niche models^12^, use of climatic refugia to shelter species of conservation concern^13^, or the implementation of resilience-oriented ecosystem management^14^. However, the theoretical understanding of these approaches, their trade-offs, and interactions is not sufficiently advanced to support the formulation of robust and consistent conservation policies. A better understanding may stimulate a broader shift in governance structures and improved coordination between land managers, politicians, and conservation organisations which appears critical to the implementation of adaptive biodiversity conservation^15^.

In Europe, Key Biodiversity Areas (KBA^16^), Natura 2000 habitat network^17^, and European Primary Forests (EPF^18^) represent examples of biodiversity conservation initiatives focused on preserving geographically well-defined areas of interest. Under climate change, some of these areas and associated conservation goals may be subject to greater climatic exposure than others. At the same time, conservation opportunities may emerge elsewhere due to future spatial or temporal variation of climate change effects. Focusing on areas with predicted stable climate, *relative to background climate change*, is increasingly recognised as a viable conservation strategy^19–21^. These climatic refugia may arice at multiple spatial scales, from small-scale poleward-facing slopes, sites affected by cold groundwater or deep snow drifts^22^, to the continental-scale refugia such as the Boreal zone in Europe^6^. Descriptions of species migration and persistence in climatic refugia during glacial and interglacial periods may be applicable to present-day conservation effort^19^, if future climate can be resolved at an appropriate scale. Climate models, which have an increasing ability to resolve the fine-scale atmospheric processes relevant for local and regional resource management and conservation planning, have thus become the central means for formulating forward-looking adaptation policies^23^.

We investigate how future climate change may affect present-day nature conservation initiatives in Europe, by considering spatial coverage and temporal persistence of regions with significantly lower or higher climatic stability. This information may be vital for the transition from the current static conservation framework to climate-adapted conservation strategies deemed necessary to avoid the imminent biodiversity breakdown^24^. At continental and regional scales, we confront climatic stability patterns with established biodiversity conservation initiatives, ranging in size from the Global Biodiversity Hotspots to small patches of primary forests. Representing the diversity of spatial scales at which atmospheric and biological processes operate^25^, we (i) evaluate the patterns of projected climatic stability in Europe at two scales; (ii) indicate some of the existing biodiversity conservation sites exposed to future high or low risk of climatic stability, and (iii) discuss how the identification of such sites can inform the formation of a climate-adapted biodiversity conservation strategy for Europe.

## Results

Our analysis of future climatic stability is based on projected changes of nine climate variables, selected to proxy key aspects of terrestrial ecosystem dynamics (Table 1). The cumulative change of all nine climate variables, termed Aggregate Climate Change (ACC), is used to identify regions of significantly higher or lower climatic stability (Getis-Ord Gi* p-value < 0.05) across Europe. We carry out this assessment at the continental and regional scales. While the continental assessment reports the large-scale latitudinal and orographic patterns of climatic stability (Eq. (1), Fig. S3), the regional assessment explores the residual ACC variation that remains after the extraction of the large-scale continental trend (Eq. (5), Fig. S3). We aim to identify and interpret climatic features likely to persist in their location throughout the twenty-first century (i.e. in periods 2021–2040, 2041–2060, and 2061–2100) and are supported by a majority of climate projections considered in this study. Areas of persistently low ACC are henceforth interpreted as potential climatic refugia and we investigate their overlap with existing biodiversity conservation initiatives.

**Table 1:** Climate variables used as predictors of future climate stability, all calculated on annual basis (*T* temperature, *Pr* precipitation, *MCMT* mean coldest month temperature).

ID	Abbreviations	Description	Units	Calculation
1	MWMT	Mean warmest month temperature	°C	$MWMT=\text{max}({T}_{mon.mean})$
2	DDa5	Degree-days above 5 °C	°C	$$ DDa5 = \sum _{{doy}} \left\{ {\begin{array}{*{20}l} {T - 5\,^{ \circ } {\text{C}},} \hfill & {T > 5\,^{ \circ } {\text{C}}} \hfill \\ {0\,^{ \circ } {\text{C}},} \hfill & {T < 5\,^{ \circ } {\text{C}}} \hfill \\ \end{array} } \right\} $$
3	FFP	Longest frost-free period	Days	The length of the longest period of consecutive days with daily minimum temperature above 0 °C
4	EQ	Ellenberg climatic quotient	°C mm^−1^	$EQ=\frac{MWMT}{{Pr}_{ann}}\times 1000$
5	Cont	Gorczynski climatic continentality	–	$Cont=\left(1.7\times \frac{MWMT-MCMT}{\text{sin}\varphi }\right)-20.4$
6	NDP	Number of dry periods	–	$$DryDay=\left\{\begin{array}{c}1, Pr < 5 {\text{mm}}\\ 0, Pr>5 {\text{mm}}\end{array}\right\}$$ *DryPeriod*: period of at least 10 consecutive DryDays *numDryPeriods:* the number of DryPeriods
7	LLDP	Length of the longest dry period	Days	Number of dry days of the longest *DryPeriod*
8	T	Annual mean temperature	°C	Average of daily mean temperatures
9	P	Annual total precipitation	mm	Sum of daily precipitations

### Continental-scale assessment

**Figure 1:**
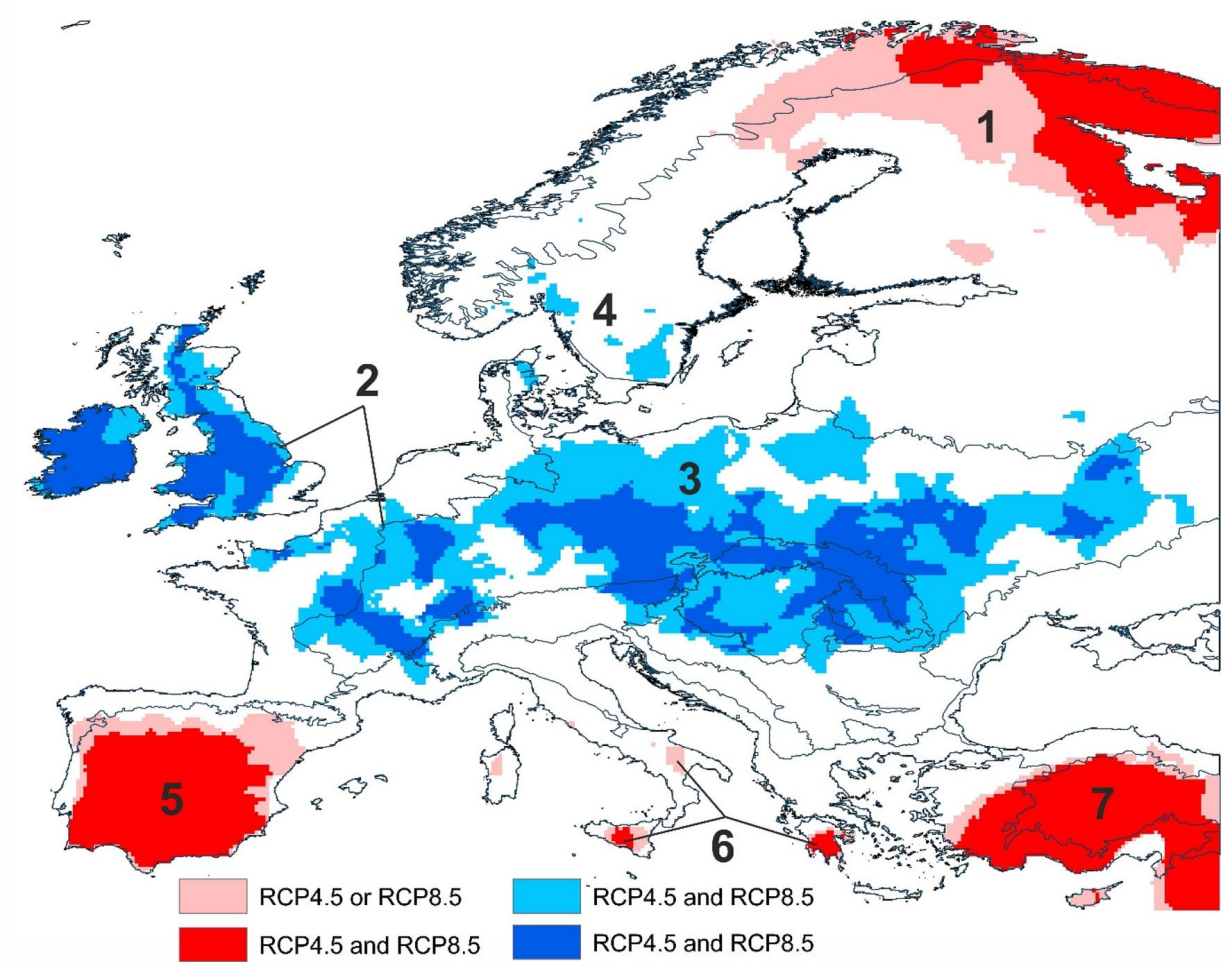
Continental-scale zones with significantly higher (blue) and lower (red) future climate stability, relative to background climate change. Coloured zones are likely to persist in all periods considered here (2041–2060, 2061–2080, and 2081–2100). Shading represents zones identified by either (light shade) or both (dark shade) RCP4.5 and RCP8.5 scenarios. Numbers show geographical sub-zones described in Table 1 and Fig. 2. Maps were created in R v. 4.0.4. (R Core Team, Vienna, Austria) and ArcGIS Desktop v. 10.7 (Esri, California, USA). The final layout was created in ArcGIS Desktop v. 10.7 (Esri, California, USA) and CorelDraw v. 20.1.0.707 (2018 Corel Corp.).

At this scale, future climate variation in Europe displays a distinct latitudinal pattern. This pattern is robust and consistent across all three periods and both RCP scenarios considered (Fig. 1). Temporal stability of identified climatic features and inter-model agreement were poorer at higher latitudes than in the rest of Europe (Figs. S1, S2). Continental-scale Central Zone (CZ) with the most stable future climate is located across the centre of Europe (ca 45°–55° N) and covers most of the Atlantic, Continental, and Steppic biogeographical zones (Fig. S1). The Northern and the Southern Zones located at high and low latitudes of Europe are characterised by future climate with significantly lower stability (NZ and SZ, respectively). The SZ extends over the Mediterranean and Anatolia, while the NZ takes in the Boreal and the Arctic regions.

**Figure 2:**
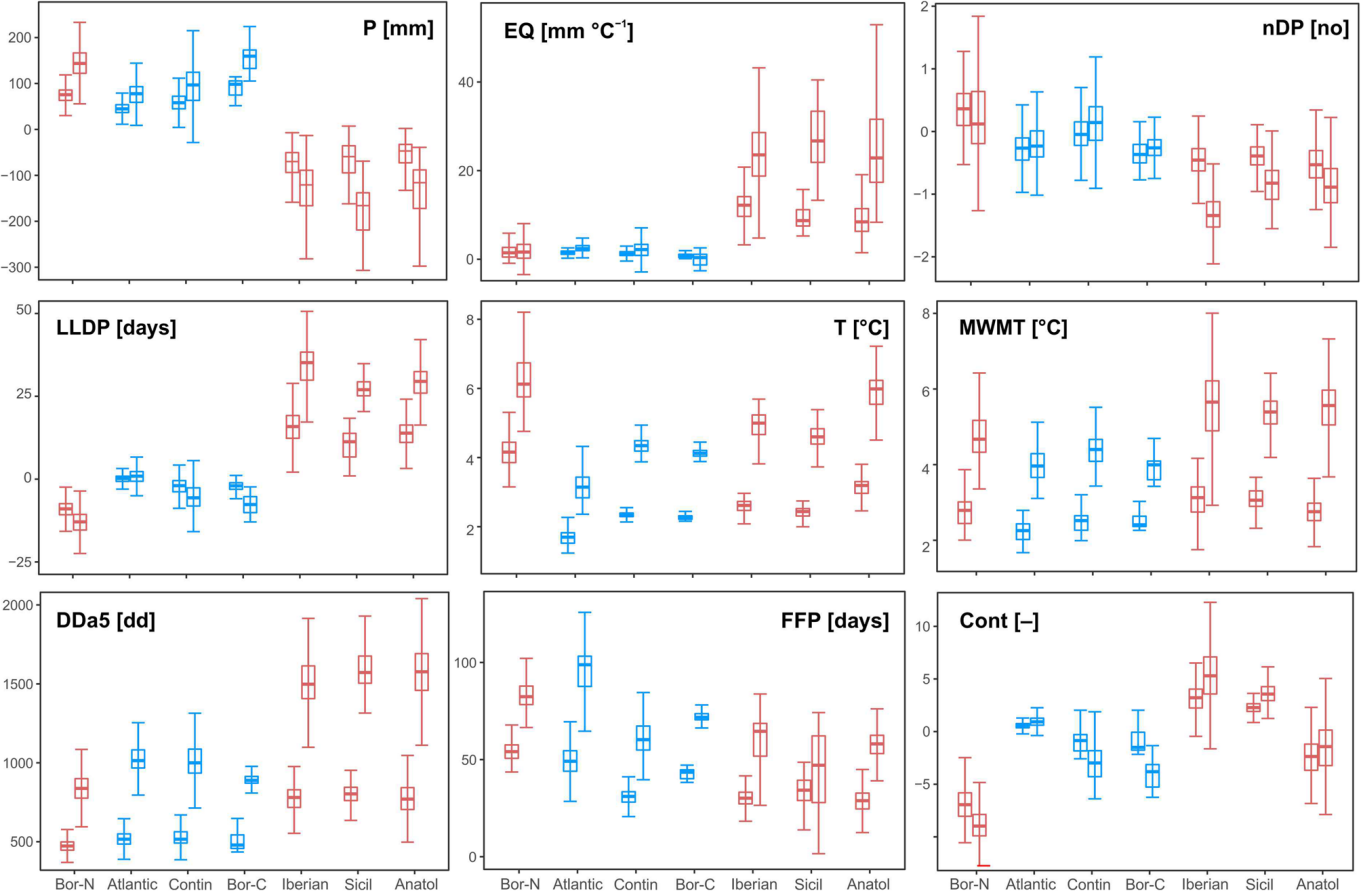
Predicted change in nine climatic variables in the geographical subzones predicted to have high or low future climatic stability (numbering of zones corresponds with Fig. 1). Boxplot pairs show projected changes driven by RCP4.5 (left) and RCP8.5 (right) and indicate their variation within each subzone. Colours indicate subzones with predicted low (red) and high (blue) climate stability. Variable acronyms: *P* annual total precipitation [mm], *EQ* Ellenberg climatic quotient [°C mm^−1^], *nDP* number of dry periods [no], *LLDP* length of the longest dry period [days], *T* annual mean temperature [°C], *MWMT* mean warmest month temperature [°C], *DDa5* degree-days above 5 °C [dd], *FFP* longest frost-free period [days], *Cont* Gorczynski climatic continentality [–]. The graphs were created in R v. 4.0.4.^26^.

The combinations of predicted changes in individual climate variables are specific to each of the major zones and their subzones (Fig. 2, Table S3). For example, the magnitude of ACC in the CZ is around 51–55% of the maximum permissible change (i.e. change that would occur if the largest change in all underlying climate variables predicted across Europe were to occur in the same pixel), whereas that predicted for the NZ or the SZ is between 65 and 75% (Table 2).

**Table 2:** Relative aggregate climate change (ACC%) in zones of low and high climatic stability as indicated by continental-scale assessment.

Code	Zone	Sub-zone	ACC%—RCP4.5	ACC%—RCP8.5	Climate stability
1	NZ	Boreal	74.3	64.4	Low
2	CZ	Atlantic	55.3	51.4	High
3		Contin	53.2	50.9	High
4		Boreal	61.6	53.5	High
5	SZ	Iberian	75.0	75.7	Low
6		Sicilian	70.4	70.8	Low
7		Anatolian	74.0	72.5	Low
8		Step	60.4	–	High

The position of zones and sub-zones and the numerical codes correspond to Fig. 1.

*NZ* Northern Zone, *CZ* Central Zone, *SZ* Southern Zone.

Looking at zones of low climatic stability predicted under RCP 8.5, the most distinctive features of the NZ are increased precipitation (+ 143 mm, 25%), shorter dry periods (− 9 days), longer frost-free periods (+ 80 days), and a strong temperature increase (+ 5.9 °C). The most striking climate features driving low climate stability in the SZ are the severe decrease of precipitation (− 153 mm, − 24%), and accompanying increases of the Ellenberg climate quotient (+ 21 to 23 mm °C^−1^) and of growing degree-days (+ 1484 to 1562 dd). Projected change in climatic continentality is another feature that underlies the difference between NZ and SZ. The patterns of change projected under RCP4.5 are similar to RCP8.5, however predicted changes are less pronounced (Fig. 2, Table S3).

### Regional-scale assessment

**Figure 3:**
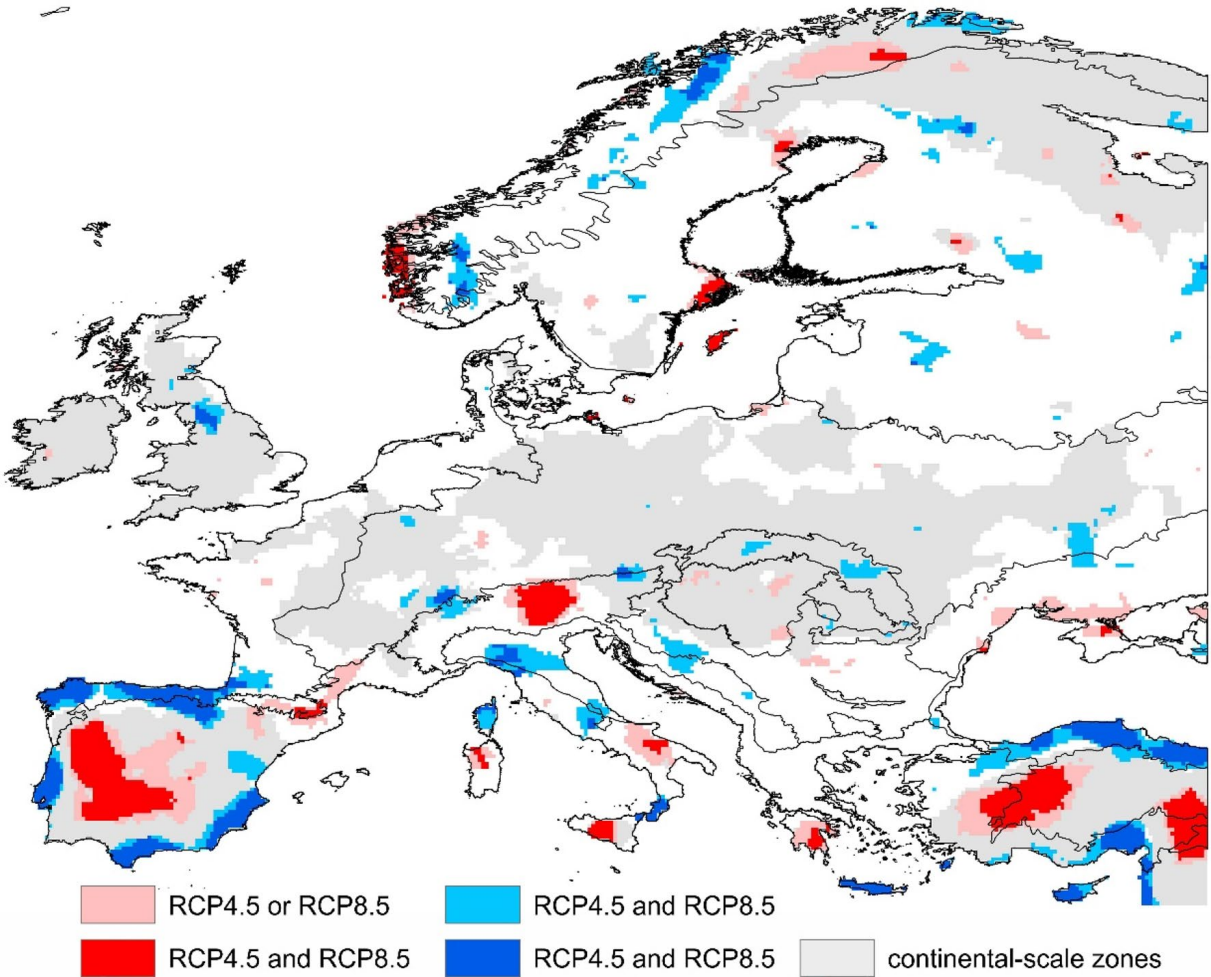
Regionally relevant zones of low (red) and high (blue) future climate stability in Europe, relative to background climate change. Coloured zones are likely to persist in all periods considered here (2041–2060, 2061–2080, and 2081–2100). Shading represents zones identified by either (light shade) or both (dark shade) RCP4.5 and RCP8.5 scenarios. Grey areas indicate continental scale for reference (Fig. 1). Maps were created in R v. 4.0.4.^26^ and ArcGIS Desktop v. 10.7 (Esri, California, USA). The final layout was created in ArcGIS Desktop v. 10.7 (Esri, California, USA) and CorelDraw v. 20.1.0.707 (2018 Corel Corp.).

Regional-scale variation of ACC is revealed by the subtraction of the continental-scale change and creates a mosaic of areas with significantly low and high climatic stability distributed all over Europe (Fig. 3). In contrast to the continental scale, the regional climatic features show poorer temporal stability and inter-RCP agreement (Fig. S2). However, a number of areas characterised by residual ACC significantly different from background persists throughout the twenty-first century under the most conservative assessment (areas identified by four out of five RMS in each RCP, Table S3).

At this scale, the most distinct areas with low climatic stability are located within the continental SZ (Fig. 1), emphasising the high regional variability of ACC in the Mediterranean. Looking at the central band identified as stable at the continental scale (CZ, Fig. 1), Eastern Alps and parts of Norway now show up as areas with regionally low climate stability. Crucially, some areas with low and high climatic stability are not too distant, e.g. seashore to inland transitions or elevation differences in mountain ranges, generating a pattern of climatic exposure potentially complicating future conservation effort.

### Spatial coincidence of conservation priorities and climate stability areas

**Figure 4:**
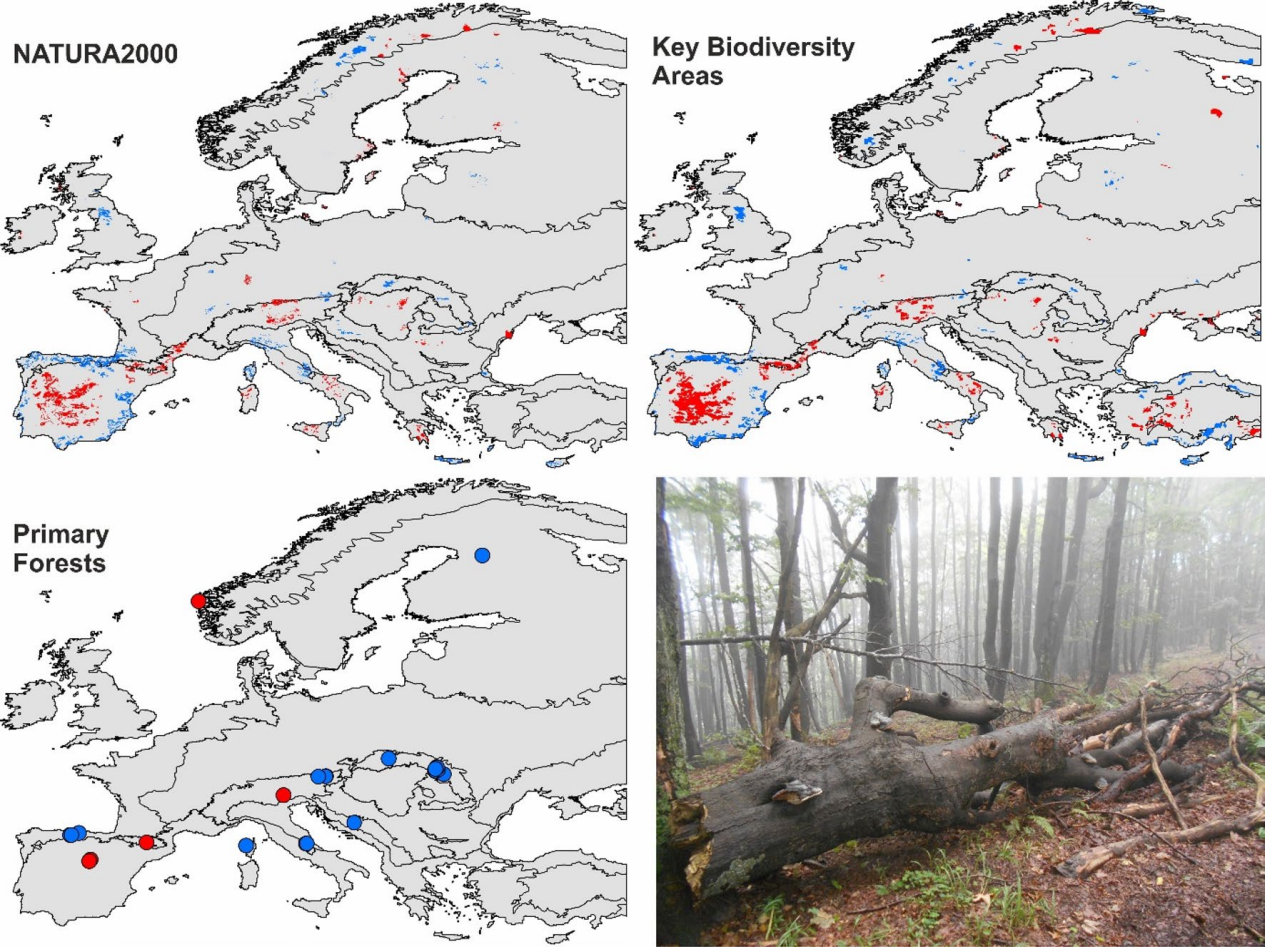
Geographical coverage of existing biodiversity conservation within areas of low (blue) or high (red) regional-scale climate stability persisting throughout the twenty-first century. Maps were created in ArcGIS Desktop v. 10.7 (Esri, California, USA). The final layout was created in ArcGIS Desktop v. 10.7 (Esri, California, USA) and CorelDraw v. 20.1.0.707 (2018 Corel Corp.). The photo illustrates a primary beech forest in the Western Carpathians, Kremnické Mts., Slovakia. Credit: Merganičová, K.

To consider the long-term implications of contrasting climatic stability for nature conservation efforts in Europe, we confront the coverage of prominent biodiversity conservation networks (Fig. 4, Supplementary Fig. S4) with continental and regional areas of contrasting climate stability persisting between 2040 and 2100.

Our predictions indicate a high probability of a significant continental-scale *northern zone* of low climatic stability covering northern Finland and Karelia (Fig. 1). This trend represents a clear threat to the Natura 2000 and KBA areas present in northern Finland. Currently, only one forest has been identified as EPF in the country, not allowing us to draw conclusions concerning this network. Looking at the predictions analysed at the regional scale, it is possible to identify several current biodiversity sites likely to fall under more stable future climate conditions than others (Fig. 4). Specifically, our data indicate that Natura 2000 and KBA sites in central Norway are significantly less exposed than those straddling the border between Norway and Finland.

**Figure 5:**
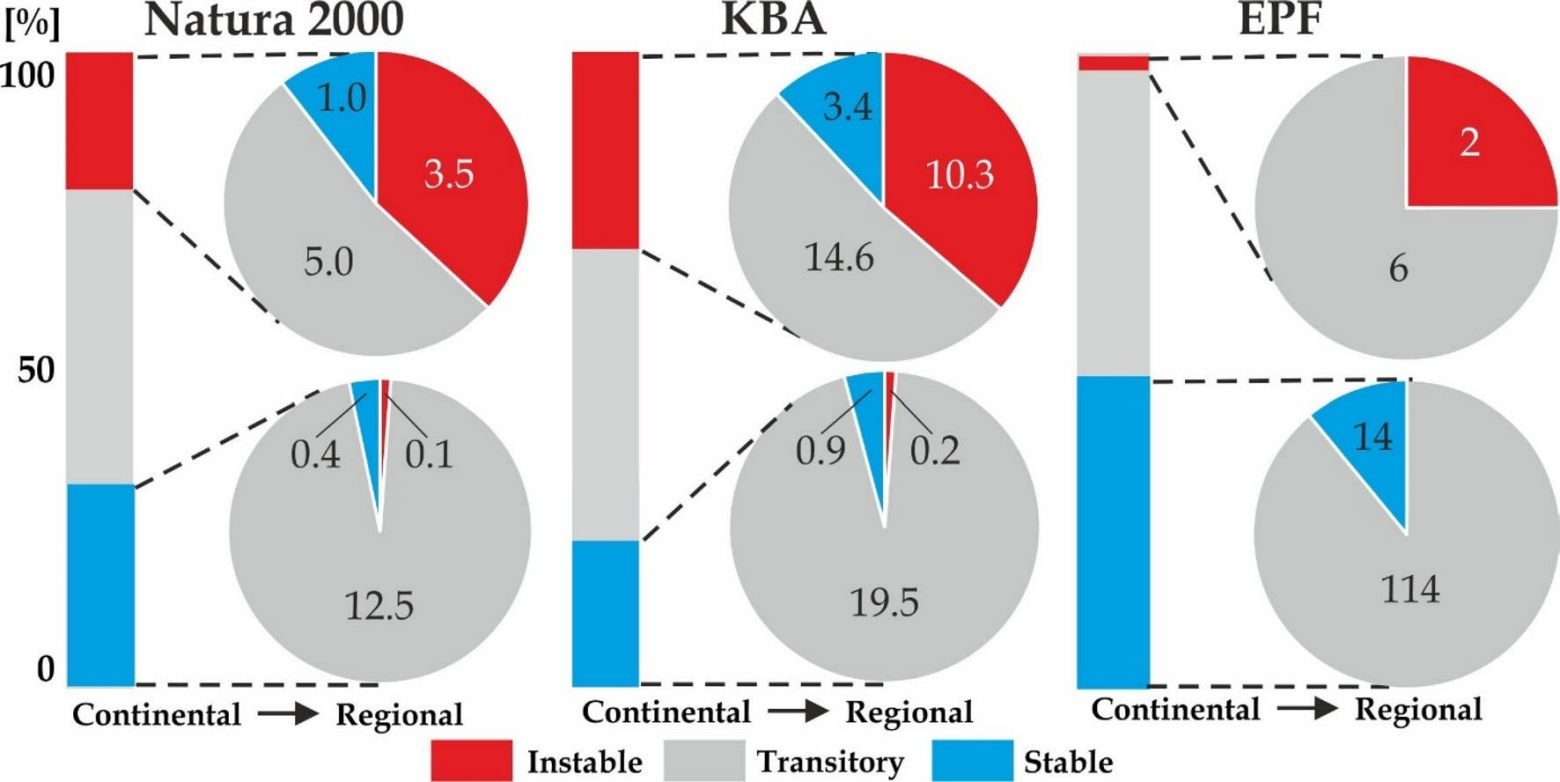
The proportion of Natura 2000, Key Biodiversity Areas, and European primary forests present in areas identified as zones of low and high climatic stability, relative to background climate change. Modelling was carried out at a continental (bars) and regional (pie chats) scales. Values in pie charts indicate areas of Natura2000 and KBA sites (mil. of ha), or the number of EPF in each climate stability category. Climatic stability as predicted under RCP4.5 or RCP8.5 and persisting throughout 2041–2100. Graphs were created in R v. 4.0.4.^26^. The final layout was created in CorelDraw v. 20.1.0.707 (2018 Corel Corp.).

Our continental-scale assessment shows that the *central zone* area of Europe stretching from the British Isles in the west to the Ukrainian steppe in the east is likely to experience significantly weaker climate change than the rest of the continent (Fig. 2, Table S3). Regional-scale assessment however shows that the greatest share of the KBA extent under future low stability climate is in the Pannonian, Steppic, Mediterranean and Anatolian zones (see Fig. S3 for the position of biogeographical zones). The above-average share of the KBA sites in zones with high climatic stability is in the Alpine North and Atlantic zones, the Arctic, and the Black Sea zones; in the latter two zones, however, the total area of the KBA sites is only minor (Fig. 5). The Alpine zone includes 119 out of 257 primary forests currently described in Europe, a continental-scale assessment shows that 100% of these are in an area with predicted low climate stability. However, the finer-scale modelling shows that only 11% of these sites are threatened by significantly low stability climate.

The *southern zone* shows the lowest climatic stability in Europe at the continental scale and largely overlaps with Europe’s prominent global biodiversity hotspot—the Mediterranean Basin. It also covers all European parts of the Irano-Anatolian and the Caucasus zones (Fig. S6). The SZ thus affects all global biodiversity sub-hotpots embedded within the Mediterranean Basin: the Rif-Betique range, Maritime and Ligurian Alps, Tyrrhenian Islands, southern and central Greece, Crete, southern Turkey and Cyprus^27^. Our model prediction at this scale thus delivers a very bleak message for biodiversity conservation sites in and around the Mediterranean.

## Discussion

Rapid and accelerating biodiversity loss due to climate change and other anthropogenic factors^28,29^ has highlighted the vulnerability of the current conservation effort, which historically did not take account of projected climate change^9^. Some areas of high biodiversity value in Europe are under increasing threat^30^, the update of their conservation strategies requires location-specific information on future viability^31^. Whilst the conservation of biodiversity and its planning is clearly important at the global scale^32,33^, most species are composed of local populations, the survival of which is driven by local climate and environment variation^34^. Emerging capability of climate models to resolve small-scale atmospheric processes allows for the identification of refugial features within the landscape^35^, offering new opportunities for confronting conservation planning with future climate stability.

### Continental scale

According to our continental-scale predictions for Europe, the most stable climate will persist in the Atlantic and Temperate zones, whilst the Mediterranean and the Boreal zones may be exposed to the least stable climate (Fig. 1), confirming existing global or continental climatic assessments^36,37^.

Although the lack of future climatic stability is similar in the northern and southern zones, the underlying changes in climatic variables are distinctly different and so are the risk factors affecting biodiversity. Increasing drought incidence at low latitudes (e.g. precipitation decreasing by > 23% and the Ellenberg quotient increasing by up to 23 mm °C^−1^; Table 1) can cause many valuable ecosystems to collapse and biodiversity to decline^38^. Already, the biodiversity in the Mediterranean is showing one of the strongest declines due to both climate change and land use^39^. At high latitudes, increasing temperature and precipitation may create conditions favourable for an expansion of southern species distribution, but may limit currently native species, and increase habitat fragmentation^40,41^. Climatic changes indicative of predicted trends have already been recorded, both in the Mediterranean and in the Boreal zone^42^, with corresponding changes in biodiversity^7^.

The middle latitudes of Europe are characterised by a highly stable climate, relative to the Europe-wide climate change. The values of ACC are lower by about 20% when compared to the northern and southern zones. Out of the three biodiversity initiatives evaluated in this study, it is the conservation of European primary forests that can benefit from this pattern. These are dominated by very long-lived organisms which benefit from stable climate and are largely distributed in this area of Europe^18^.

### Regional scale

The continental-scale climate change pattern may inform strategic conservation planning but may not be practical for guiding nature conservation efforts on the ground. In Europe, this relies on networks of protected areas of varying size. To illustrate the potential to aid conservation decision-making, we identified smaller-scale areas with significantly low and high climatic stability. While most of the regional areas are ephemeral (i.e., predicted climate change did not persist during the three investigated periods), some have high temporal persistence and are consistently predicted under a majority of climate projections. Smaller-scale refugia with stable climate exist even within the climatically highly exposed Mediterranean and Boreal regions, providing interesting opportunities for biodiversity conservation there. The largest regional areas of stable climate are predicted for low latitudes, while their counterparts at middle and high latitudes are smaller and more fragmented.

Interpreting the pattern of regional zones of stability is fraught with difficulty, a large number of contributing variables plays a role: different representations of atmospheric processes in used climate models, and rather complex procedures used to identify them. For example, some of the most significant areas of stable climate were identified close to the coast in the Mediterranean and Anatolian zones, suggesting the importance of land–ocean interface in moderating the regional climate^19^. Still, further research is required to understand processes forming the regional refugia.

### Implications for biodiversity conservation

Our analysis indicates conservation initiatives at risk and illustrates how some localities within a specific region may be better placed to withstand climate change than others. Europe is characterised by long-established land use and development process, regional climate stability maps may be used to move towards adaptive biodiversity conservation strategy. For example, rules can be put in place and interventions triggered when pre-determined climatic conditions identified by modelling are reached^43^, alternatively climate stability indication may guide current re-naturalisation efforts^44^. A potentially useful outcome could be a mosaic of areas with relatively stable future climate to serve as refugia or steppingstones aiding migration^45^. Identification of areas most exposed to future climate change may then stimulate vulnerability studies and aid the targeting of adaptation effort and investment^46^.

We found that the three prominent European biodiversity initiatives considered here overlap with areas potentially serving as climate refugia to a different degree, with large geographical differences. For example, EPFs typically are small remnants of natural vegetation, a number of which is found in the stable CZ and some are within regional climate refugia. These forests may serve as targets of conservation efforts due to their better future prospects. In contrast, Natura2000 and KBA sites tend to be larger and actively managed. Here, the indication of future climate stability can be used to plan in-situ conservation (e.g. measures to reduce risk and foster resilience), or even ex-situ conservation by expanding existing areas into neighbouring areas of stable climate^47^.

A different approach may be needed in areas of high conservation value located in areas of predicted low climatic stability. Following an assessment of climate risk for a species or a habitat, a revision of the current conservation framework may include a range of interventions from abandoning the conservation effort to the deployment of active measures aimed at their restructuring and adaptation^48^.

### Limitations and future work

The literature documents interest in predicting the persistence of existing microclimates, niches or even habitats. Historically, a range of techniques has been applied to the identification of areas of high stability, with the view of informing nature conservation. The methods differ depending on the spatial scale, type of system and sub-system considered (climate, habitat, biome), or the type of perturbance (warming or cooling, droughts or flooding, fluctuating sea-levels)^19,49^. Approaches to refugia mapping include the use of topographic maps to identify regions with suitable communities^50^, combining climate change and environmental diversity velocity metrics^51^, or the integration of species traits and information on climatic exposure^52,53^.

Model-based climate change projections form the underlying dataset used in this study. Our approach copes with the complexity of predictions by integrating scenarios, variables, and time periods, but it allows for tracking of individual factors which can translate the emergent climate patterns into a useful frame of reference. However, the approach neglects the biological perspective somewhat: we partly addressed this issue by selecting climate variables and indices indicative of biological performance. A fully biology-focussed approach may be crucial for informing conservation planning. Future studies could investigate identified climate stability zones from the perspective of traits, environmental tolerance, or adaptive capacity of species, communities, or ecosystems. This may be achieved by combining our approach with Species Distribution Models or more complex process-based ecosystem models.

An arbitrary multidecadal resolution used in this study (focussing on 20-year periods) can be limiting, particularly if species and ecosystems of interest are sensitive to episodic climatic perturbations. Future studies may attempt to evaluate climate stability at an annual step, or identify the frequency of years with climatic envelope unsuitable for the target species or ecosystem. Finally, the usefulness of our findings is currently limited by the resolution and the level of process detail of current RCMs. Anticipated transition to convection-permitting modelling systems^35^ could be an important step towards improved predictions of future climate stability at regional and local scales. Future research should also aim to better understand particular physical and biological processes forming areas of stable climate, and how these processes are integrated into climate models.

## Methodology

### Biodiversity indicators

We used the distribution of global biodiversity hotspots extending into Europe as an indicator of continental-scale biodiversity value of an area. Here, global hotspots are chiefly represented by the Mediterranean basin (MB), with small incursions from the Irano-Anatolian (IA) and Caucasus (CA) hotspots^27^. To carry out a regional-scale analysis, we use data from three initiatives describing areas of high current biodiversity value in Europe (Fig. S2): Key Biodiversity Areas (KBA^16^), Natura 2000 habitat network^17^, and European Primary Forests (EPF^18^). Each initiative uses different criteria for site selection, these can be summarised as:

1. *KBA* are sites holding biodiversity elements that are globally restricted, or at risk of disappearing. KBA selection criteria integrate evaluation of biodiversity at genetic, species, and ecosystem levels. The KBA approach aims to support the identification of sites important for elements of biodiversity not considered in existing approaches. The KBA database builds on previous efforts and includes subsets of biodiversity such as birds, fungi, higher plants or butterflies.
2. *Natura 2000* is one of the tools delivering EU Biodiversity Strategy, which aims to halt the loss of biodiversity and ecosystem services on its territory. Natura 2000 is a network of core breeding and resting sites, plus some rare natural habitat types. The aim of the network is to ensure the long-term survival of Europe's most valuable and threatened species and habitats, listed under both the Birds Directive and the Habitats Directive. Natura 2000 covers both land and sea ecosystems, however this study focuses on terrestrial habitats only.
3. *EPF* is the most comprehensive ground-truthed database of currently known naturally regenerated forests of native species in Europe, with no clearly visible signs of past human activity or significant disturbance of ecological processes. Excluding Russia, large patches of primary forests no longer exist in Europe, this database thus includes forests previously classified as primeval, virgin, near-virgin, old-growth, and long-untouched, where empirical evidence suggests an absence of direct human impact for at least 200 years.

### Climate data

We used the ECLIPS (European CLimate Index ProjectionS) 1.1 climate dataset^54^, which contains long-term climatological averages for several different climate variables and indices. The data are available for five bias-corrected regional climate model simulations created within the framework of the EURO-CORDEX project^55^ and cover the whole of Europe (EUR-11 CORDEX domain) with a 0.11° × 0.11° horizontal resolution. The simulations were driven by two Representative Concentration Pathway scenarios: RCP4.5 and RCP8.5, producing 10 climate projections in total (5 RCMs × 2 RCPs). The testing of bias-corrected temperature and precipitation model results against the observation-based E-OBS data set^56^ was conducted by^57^. The dataset is available at 10.5281/zenodo.1204351.

We investigated three future time periods—2041–2060, 2061–2080 and 2081–2100. To evaluate the degree of future climatic stability, we compared the future climate against the model-based reference climate from the period 1961–1990.

We initially considered 23 climate variables as indicators of the degree of future climate stability, the list of variables was refined by inspecting their correlation matrix for redundancy. Delta values (predicted future values minus reference value) were used to generate the correlation matrix to correspond with further analysis of low climate stability which is based solely on differences between climate periods. Pearson coefficient > 0.7 was used to discard one variable out of each pair of correlated variables (see Dormann et al.^58^ for rationale). Table 1 lists the final set of variables used in this study.

### Assessment of future climatic stability

We identified areas with climatic stability significantly different from their surroundings^59^ as follows: (i) for each grid cell in climate maps, we calculated Euclidean distance between past and future climates in an n-dimensional space defined by a set of climate variables to produce a continuous map of Aggregate Climate Change (ACC); (ii) we used the resulting ACC map to identify areas with significantly low and high climatic stability using the Getis-Ord Gi* statistics; (iii), we then subtracted the continent-wide trend from the ACC data to create a residual ACC map, and inspected it using Getis-Ord Gi* to uncover regional areas with contrasting climatic stability. This analysis was conducted for all climate projections and time periods, partial results were then aggregated to identify the most robust and temporally stable areas with low (refugia) and high (hotspots) climatic stability.

#### Calculation of aggregate climate change

We first calculated Standard Euclidean Distance (SED^60^) to characterise ACC between present and future periods in a n-dimensional climate space:

1 $$ {\text{ACC}} = \sqrt {\left( {\sum\limits_{{{\text{i}} = 1}}^{{\text{n}}} {{\text{SED}}_{{\text{v}}} } } \right)} . $$

SED for the variable *v* is defined as:

2 $${\text{SED}}_{\text{v}}={\left({\Delta }_{\text{v}}/\text{max}{\left[\left|{\Delta }_{\text{v}}\right|\right]}_{\text{xy}}\right)}^{2},$$

where $\left({\Delta }_{\text{v}}\right)$ is the change in climate variable *v* at each grid point between two time periods, and $\text{max}{\left[\left({\Delta }_{\text{v}}\right)\right]}_{\text{xy}}$ is the maximum value of the change in variable *v* over the entire study area. In our study, ${\Delta }_{\text{v}}$ is calculated for each pixel and each variable as the difference between a future period (2021–2040, 2041–2060, and 2061–2100) and the reference period (1961–1990). This analysis was conducted separately for 10 climate projections and 3 future time periods (i.e. 30 ACC maps were produced).

To prevent the undesired effect of using $\text{max}{\left[\left({\Delta }_{\text{v}}\right)\right]}_{\text{xy}}$ to standardize the ${\Delta }_{\text{v}}$, which can be affected by a single extreme value, we used the 95% quantile instead:

3 $$ {\text{SED}}_{{\text{v}}}  = \left( {\Delta _{v}^{'} /{\text{Q}}_{{95}} \left[ {\left( {\Delta _{{\text{v}}} } \right)} \right]_{{{\text{xy}}}} } \right)^{2} \left\{ {\begin{array}{*{20}l}    {if\,SEDv > 1\,then\,SEDv = 1} \hfill  \\    {if\,SEDv < 1\,then\,SEDv = SEDv} \hfill  \\   \end{array} } \right\}. $$

Raw ACC values were expressed as a percentage of the maximum permissible change to improve the clarity and interpretation of the assessment. The maximum change is defined as the square root of the number of variables used for the ACC calculation (*n* = 9 in this study):

4 $${\text{ACC}}_{\text{\%}}=\frac{\text{ACC}}{\sqrt{\text{n}}}\times 100.$$

While this type of ACC calculation may inform on large-scale patterns of climatic exposure relevant at the continental scale, it may obscure small-scale patterns of regional importance. We also carried out a regional-scale study where patterns of climatic stability are identified from residual ACC_%_ generated by subtracting the continental-scale trend from raw ACC_%_ values. The Europe-wide spatial trend to be subtracted from the ACC_%_ was defined using the following generalized additive model:

5 $${\text{ACC}}_{\text{\%}}= \propto +f\left(Lat, Long\right)+ \varepsilon ,$$

where *f* is the spline-on-the-sphere basis function^61^, *Lat* is latitude and *Long* is longitude in degrees, $\propto$ is the intercept and $\varepsilon$ is the error term.

Due to unequal goodness of fit to 30 ACC surfaces (5 RCMs × 2 RCP scenarios × 3 future time periods), we did not use the estimation of function parameters based on the automatized penalization procedure as it would hamper their comparison. Instead, we iteratively searched for the parameter *k* (number of knots of the spline) to reach 70% fit of the function to the data in all ACCs, with a minor tolerance. All calculations were conducted in R 342 v.3.3.2^26^ using the mgcv v. 1.8–23 library^62^.

#### Getis-Ord Gi* statistics

We used a rigorous approach based on the Getis-Ord Gi* statistics to identify discreet zones with significantly low and high climate stability both at continental (based on the original ACC) and regional (based on the residual ACC) scales. The procedure was developed to evaluate spatial data for clustering of high and low values, often referred to as hotspots and coldspots of a given phenomenon^63^. The procedure evaluates whether the sum of values surrounding each feature (grid-cell in the current study) differs significantly from the sum calculated for the spatial domain under investigation.

6 $${G}_{i}^{*}=\frac{\sum_{j=1}^{n}{w}_{i,j}{x}_{j}-\overline{X}\sum_{j=1}^{n}{w}_{i,j}}{S\sqrt{\frac{[n\sum_{j=1}^{n}{w}_{i,j}^{2}-{(\sum_{j=1}^{n}{w}_{i,j})}^{2}]}{n-1}}},$$

where *x*_*j*_ is the ACC value for the grid-cell *j*, *w*_*i,j*_ is the weight between grid-cells *i* and *j*, and *n* is the total number of grid-cells in the investigated spatial domain. *X* and *S* are calculated as:

7 $$\overline{X}=\frac{\sum_{j=1}^{n}{x}_{j}}{n},$$

8 $$S=\sqrt{\frac{\sum_{j=1}^{n}{x}_{j}^{2}}{n}}-{\left(\overline{X}\right)}^{2}.$$

*w*_*i,j*_ is calculated based on the conceptualized spatial relationship between samples. We used inverse distance weighting to attach a larger influence to grid cells nearer to the target grid-cell compared to more distant grid-cells. This procedure generates *z*-values and thus allows for testing of the statistical significance of identified clusters. We used α = 0.05 to identify clusters of grid-cells significantly different from the surrounding area. To account for the effects of multiple testing and spatial dependency of the data, a correction for the False Discovery Rate was applied^64^. The analysis was conducted in ArcGis (Release 10.8. Redlands, CA).

#### Post-processing

The final evaluation constructed 30 categorical maps of areas with significantly low and high climatic stability obtained using the Getis-Ord Gi* statistics; these maps correspond to 5 RCMs driven by two RCP scenarios, and 3 future time periods. These maps were combined using a set of criteria to identify the final locations of key continental or regional areas with high or low climatic stability. First, the key areas had to be identified by at least four out of the five RCMs used here, relative to each RCP scenario. Second, we evaluated whether the locations of key areas were identified under a single or both RCP scenarios. Third, we evaluated the temporal persistence of the identified key areas. Concerning the needs of future conservation planning, we opted to use a conservative approach and considered only those areas, which persisted in their location throughout the three future time periods.

We used the biogeographical zones of Europe^65^ (Fig. S3) to categorize the identified key areas with respect to current biogeographical conditions in Europe. The analyses were conducted using the R 342 v.3.3.2^26^ using raster^66^, and rgdal^67^ libraries.

## Supplementary Information

Supplementary Information.
