# Supplementary material for: Fine-scale variation in projected climate change presents opportunities for biodiversity conservation in Europe
Source: Sci Rep. 2021 Aug 26;11:17242. doi: 10.1038/s41598-021-96717-6 (PMC8390652; doi:10.1038/s41598-021-96717-6)
Supplement: Supplementary file 1 — Supplementary Information. [file 41598_2021_96717_MOESM1_ESM.docx]

**Supplementary information A (tables and figures)**

**Fine-scale variation in projected climate change presents opportunities for biodiversity conservation in Europe**

Tomáš Hlásny^1^, Martin Mokroš^1^, Laura Dobor^1^, Katarína Merganičová^1^, Martin Lukac^1,2 *^

^1^Czech University of Life Sciences Prague, Faculty of Forestry and Wood Sciences, Kamýcká 129, 165 2

^2^School of Agriculture, Policy and Development, University of Reading, Reading, RG6 6AR, UK

**Supplementary Table S1.** Bias-corrected CORDEX simulations used in this study.

| **Model ID** | **Institute** | **RCM** | **Driving GCM** |
| --- | --- | --- | --- |
| 1. | Climate Limited-area Modelling Community (CLM-Community) | CLMcom-CLM4-8-17 | CNRM-CERFACS-CNRM-CM5 |
| 2. | Climate Limited-area Modelling Community (CLM-Community) | CLMcom-CLM4-8-17 | MPI-M-MPI-ESM-LR |
| 3. | Danish Meteorological Institute (DMI) | DMI-HIRHAM5 | ICHEC-EC-EARTH |
| 4. | Royal Netherlands Meteorological Institute (KNMI) | KNMI- RACMO22E | MOHC-HadGEM2-ES |
| 5. | Helmholtz-Zentrum Geesthacht, Climate Service Center, Max Planck Institute for Meteorology (MPI-CSC) | MPI-CSC-REMO2009 | MPI-M-MPI-ESM-LR |

**Supplementary Table S2.** The extent of biodiversity sites in each of the biogeographical zones of Europe and the European total. EPF – European Primary Forests ^16^, KBA – Key Biodiversity Areas ^14^, Natura 2000 – Natura 2000 habitat sites ^15^. The map of biogeographical zones is shown in Supplementary Figure S2.

| **Biogeographical zone** | **Latitudinal zone** | **KBA  [thousands ha]** | **Natura2000 [thousands ha]** | **EPF  [number]** |
| --- | --- | --- | --- | --- |
| Arctic | North | 136 | 0 | 0 |
| Boreal |  | 9 516 | 3 413 | 34 |
| Alpine North |  | 3 072 | 2 362 | 3 |
| Atlantic | Centre | 6 552 | 5 867 | 12 |
| Continental |  | 23 191 | 12 858 | 58 |
| Pannonian |  | 2 263 | 1 502 | 3 |
| Alpine |  | 9 592 | 6 299 | 119 |
| Steppic |  | 2 105 | 566 | 0 |
| Mediterranean | South | 32 664 | 12 981 | 28 |
| Anatolian |  | 2 502 | 0 | 0 |
| BlackSea |  | 1 213 | 447 | 0 |
| **Europe** |  | **92 806** | **46 295** | **257** |

**Supplementary Table S3.** Aggregate Climate Change (ACC%) and predicted changes of nine contributing variables in the geographical subzones of the three continental zones considered here (North, Central and South) displaying significantly high or low future climatic stability (geographical position of subzones is indicated in the Supplementary Figure 1). ACC% indicates the relative magnitude of the Aggregate Climate Change within each subzone (100% indicates cases where all underlying climate variables reach their continent-wide maximum). Individual climate variable data show predicted change from 1961-1990 to 2081-2100 and are calculated as a mean of five regional climate model outputs driven by RCP4.5 and RCP8.5. Variable description: ACC% – Aggregate Climate Change, P – annual total precipitation [mm], EQ – Ellenberg climatic quotient [°C mm^-1^], nDP – number of dry periods, LLDP – length of the longest dry period [days], T – annual mean temperature [°C], MWMT – mean warmest month temperature [°C], DDa5 – degree-days above 5°C [°C], FFP – longest frost-free period [days] and Cont – Gorczynski climatic continentality. Colour shading indicates the direction and the magnitude of predicted change. The sub-zones were used in the current study to support the interpretation of model results and carry no further biogeographical meaning.

| ID | Zone | Sub-zone | ACC [%] | P [mm] | EQ [mm °C^-1^] | nDP [no] | LLDP [days] | T [°C] | MWMT [°C] | DDa5 [dd] | FFP [days] | Cont [–] |
| --- | --- | --- | --- | --- | --- | --- | --- | --- | --- | --- | --- | --- |
|  |  |  | Precipitation-related variables  (RCP4.5) | | | | | Temperature-related variables (RCP4.5) | | | | Other |
| 1 | NZ | Boreal | **74.3** | 73.2 | 1.0 | 0.2 | -7.2 | 4.0 | 2.7 | 486.0 | 53.3 | -7.2 |
| 2 | CZ | Atlantic | **55.3** | 46.9 | 1.6 | 3.0 | 0.3 | 1.9 | 2.4 | 556.0 | 44.7 | 0.3 |
| 3 |  | Contin | **53.2** | 58.8 | 0.9 | -0.1 | -0.9 | 2.4 | 2.5 | 549.7 | 37.8 | -0.9 |
| 4 |  | Boreal | **61.6** | 91.8 | 0.1 | -0.2 | -3.4 | 2.7 | 2.2 | 448.0 | 46.0 | -3.4 |
| 5 | SZ | Iberian | **75.0** | -75.2 | 11.1 | -0.4 | 2.6 | 2.5 | 2.9 | 751.4 | 29.8 | 2.6 |
| 6 |  | Sicilian | **70.4** | -66.8 | 6.9 | -0.3 | 0.3 | 2.6 | 2.8 | 759.8 | 29.9 | 0.3 |
| 7 |  | Anatolian | **74.0** | -59.7 | 7.5 | -0.6 | -3.5 | 3.2 | 2.7 | 717.4 | 28.1 | -3.5 |
| 8 |  | Step | **60.4** | 3.3 | 4.1 | -0.2 | -0.5 | 2.6 | 3.0 | 656.0 | 27.8 | -0.5 |

| ID | Zone | Sub-zone | ACC [%] | P [mm] | EQ [mm °C^-1^] | nDP [no] | LLDP [days] | T [°C] | MWMT [°C] | DDa5 [dd] | FFP [days] | Cont [–] |
| --- | --- | --- | --- | --- | --- | --- | --- | --- | --- | --- | --- | --- |
|  |  |  | Precipitation-related variables  (RCP8.5) | | | | | Temperature-related variables (RCP8.5) | | | | Other |
| 1 | NZ | Boreal | **64.4** | 142.3 | 0.6 | -0.2 | -8.8 | 5.9 | 4.4 | 850.1 | 80.7 | -8.8 |
| 2 | CZ | Atlantic | **51.4** | 79.6 | 2.4 | -0.3 | 0.4 | 3.4 | 4.2 | 1064.4 | 92.0 | 0.4 |
| 3 |  | Contin | **50.9** | 88.1 | 2.0 | 0.0 | -2.8 | 4.3 | 4.4 | 877.2 | 72.6 | -2.8 |
| 4 |  | Boreal | **53.5** | 153.6 | 0.1 | -0.4 | -3.7 | 4.3 | 4.0 | 877.2 | 72.6 | -3.7 |
| 5 | SZ | Iberian | **75.7** | -136.5 | 23.3 | -1.2 | 4.9 | 4.9 | 5.7 | 1484.2 | 57.9 | 4.9 |
| 6 |  | Sicilian | **70.8** | -178.1 | 21.8 | -0.6 | 1.3 | 5.2 | 5.4 | 1561.8 | 58.2 | 1.3 |
| 7 |  | Anatolian | **72.5** | -144.2 | 22.7 | -1.0 | -2.7 | 6.2 | 5.7 | 1512.3 | 57.9 | -2.7 |
| 8 |  | Step | **—** | — | — | — | — | — | — | — | — | — |

**
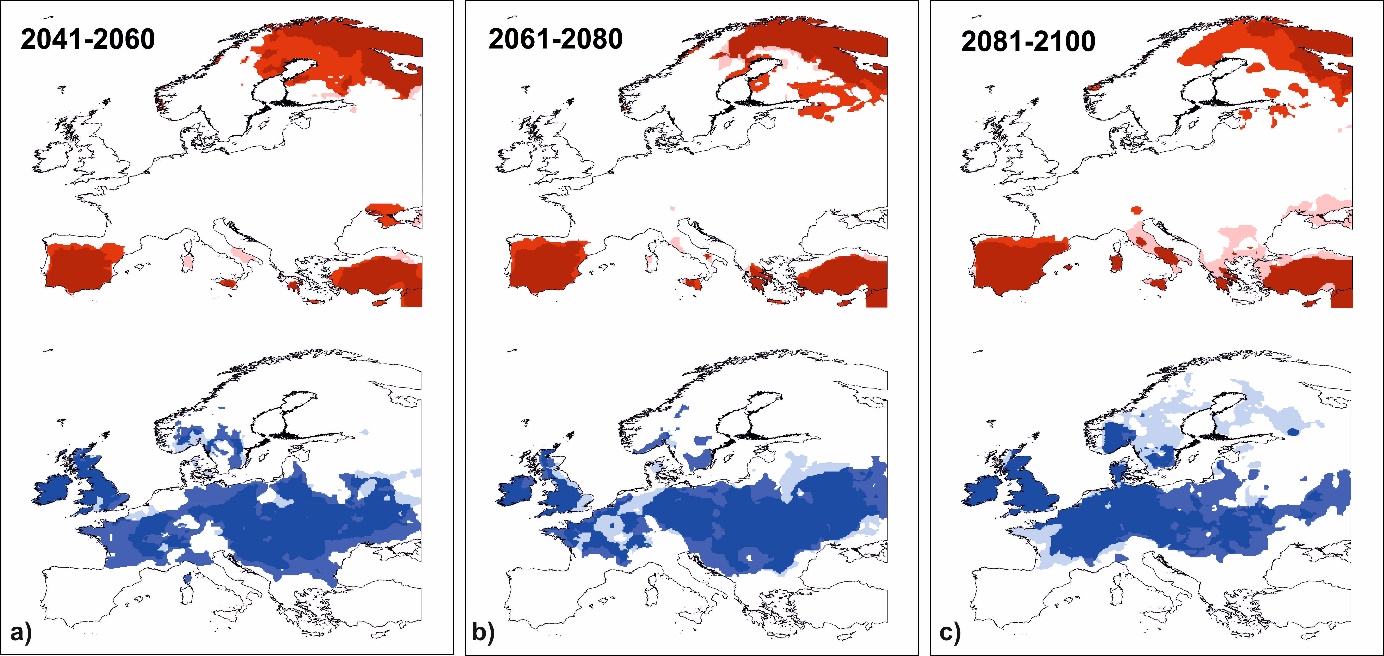
**

**Supplementary Figure S1** Predicted zones with significantly low (red) and high (blue) climatic stability in Europe, relative to continental background climate change. Indication of climate stability zones during three separate time periods. The maps were created in R v. 4.0.4. (R Core Team, Vienna, Austria). The final layout was created in ArcGIS Desktop v. 10.7 (Esri, California, USA) and CorelDraw v. 20.1.0.707 (2018 Corel Corp.).

**
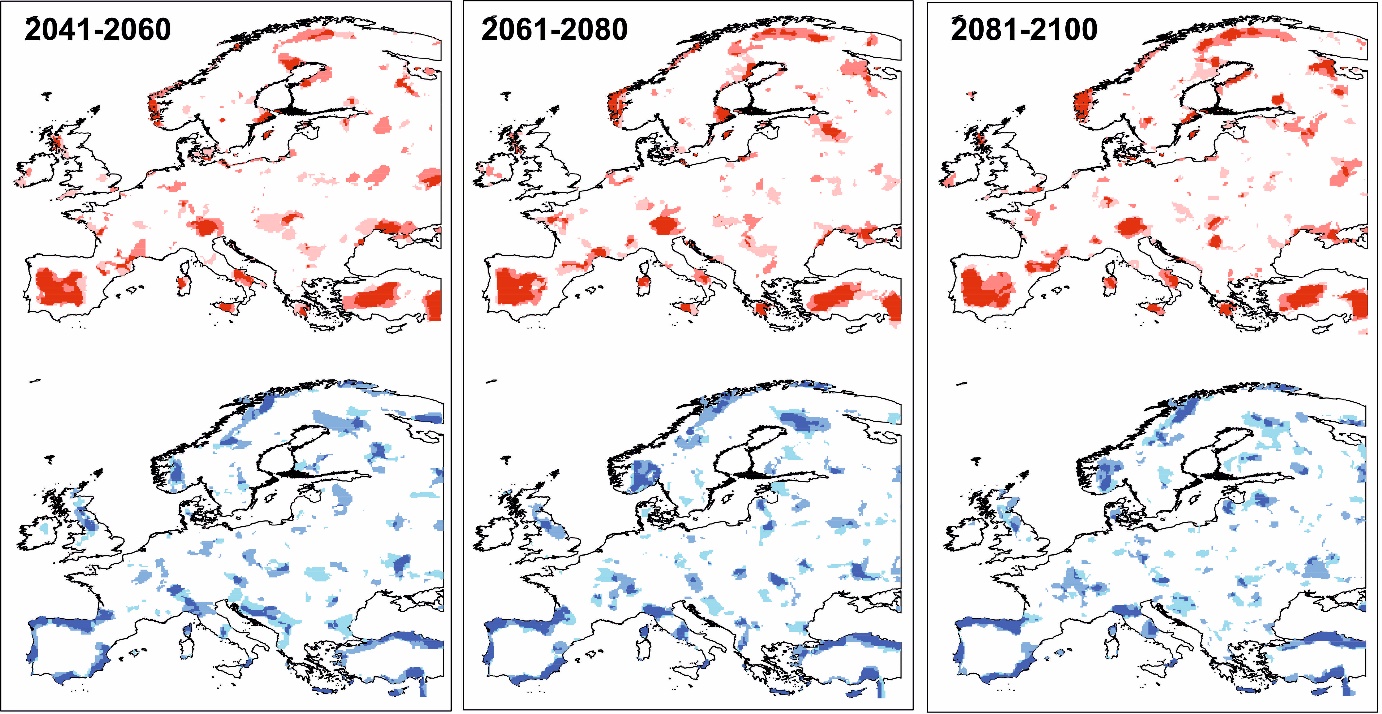
**

**Supplementary Figure S2** Predicted zones with significantly low (red) and high (blue) climatic stability in Europe, relative to regional background climate change. Indication of climate stability zones during three separate time periods. The maps were created in R v. 4.0.4. (R Core Team, Vienna, Austria). The final layout was created in ArcGIS Desktop v. 10.7 (Esri, California, USA) and CorelDraw v. 20.1.0.707 (2018 Corel Corp.).

**Supplementary Figure S3.** Geographically distinct sub-zones of interest identified at the continental scale and typical for either high or low climatic stability. Climatic characteristics and the ID number of each sub-zone are described in Supplementary Table S3. The maps were created in R v. 4.0.4. (R Core Team, Vienna, Austria). The final layout was created in ArcGIS Desktop v. 10.7 (Esri, California, USA) and CorelDraw v. 20.1.0.707 (2018 Corel Corp.).

**
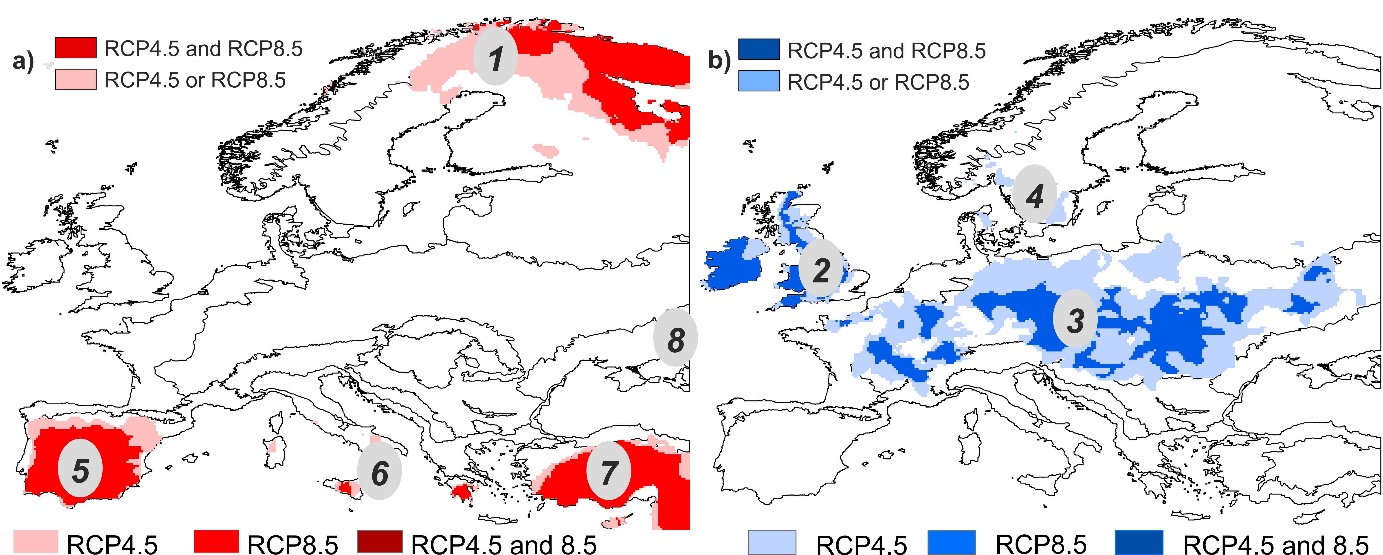
**

**Supplementary Table S4.** The proportion of the area of KBA (Key Biodiversity Area) and Natura 2000 sites or the number of EPF (European Primary Forests) likely to experience significantly low or high climatic stability, compared to their surroundings. Blank cells denote no sites present in certain biogeographic zones.

| Continental-scale assessment | | | | | | | | | |
| --- | --- | --- | --- | --- | --- | --- | --- | --- | --- |
| Zone | KBA | | | Natura 2000 | | | EPF | | |
|  | Low stability | Transitory | High stability | Low stability | Transitory | High stability | Low stability | Transitory | High stability |
| Arctic | 0,0 | 100,0 | 0,0 | – | – | – | – | – | – |
| Boreal | 40,8 | 57,7 | 1,5 | 29,6 | 67,8 | 2,5 | 14,7 | 82,4 | 2,9 |
| Alpine North | 34,5 | 65,5 | 0,0 | 50,9 | 49,1 | 0,0 | 0,0 | 100,0 | 0,0 |
| Atlantic | 1,0 | 63,4 | 35,6 | 2,6 | 56,2 | 41,2 | 0,0 | 33,3 | 66,7 |
| Continental | 0,0 | 39,7 | 60,3 | 0,0 | 43,4 | 56,6 | 0,0 | 22,0 | 78,0 |
| Pannonian | 0,0 | 100,0 | 0,0 | 0,0 | 100,0 | 0,0 | 0,0 | 0,0 | 100,0 |
| Alpine | 0,5 | 64,2 | 35,3 | 0,3 | 58,4 | 41,3 | 0,0 | 40,0 | 60,0 |
| Steppic | 0,0 | 98,2 | 1,8 | 0,0 | 100,0 | 0,0 | – | – | – |
| Mediterranean | 64,8 | 35,2 | 0,0 | 56,7 | 42,6 | 0,7 | 11,5 | 84,6 | 3,8 |
| Anatolian | 99,8 | 0,2 | 0,0 | – | – | – | – | – | – |
| BlackSea | 16,7 | 83,3 | 0,0 | 0,0 | 100,0 | 0,0 | – | – | – |

| Regional-scale assessment | | | | | | | | | |
| --- | --- | --- | --- | --- | --- | --- | --- | --- | --- |
| Zone | KBA | | | Natura 2000 | | | EPF | | |
|  | Low stability | Transitory | High stability | Low stability | Transitory | High stability | Low stability | Transitory | High stability |
| Arctic | 0,0 | 63,7 | 36,3 | – | – | – | – | – | – |
| Boreal | 4,5 | 91,9 | 3,6 | 2,6 | 95,6 | 1,8 | 0,0 | 97,1 | 2,9 |
| Alpine North | 13,7 | 74,2 | 12,1 | 8,4 | 76,8 | 14,8 | 0,0 | 100,0 | 0,0 |
| Atlantic | 0,5 | 78,1 | 21,4 | 0,9 | 84,7 | 14,4 | 0,0 | 75,0 | 25,0 |
| Continental | 1,1 | 96,4 | 2,5 | 1,1 | 96,7 | 2,2 | 0,0 | 100,0 | 0,0 |
| Pannonian | 9,4 | 90,6 | 0,0 | 9,2 | 90,8 | 0,0 | 0,0 | 100,0 | 0,0 |
| Alpine | 14,3 | 81,2 | 4,5 | 8,9 | 86,7 | 4,4 | 1,6 | 84,8 | 13,6 |
| Steppic | 16,1 | 82,1 | 1,8 | 15,8 | 84,2 | 0,0 | – | – | – |
| Mediterranean | 29,5 | 56,0 | 14,6 | 29,2 | 55,8 | 15,0 | 7,7 | 88,5 | 3,8 |
| Anatolian | 38,7 | 55,2 | 6,1 | – | – | – | – | – | – |
| BlackSea | 8,8 | 48,7 | 42,5 | 23,9 | 62,8 | 13,3 | – | – | – |

**Supplementary Figure S4.** Geographical distribution of nature conservation sites considered in the current study. Biogeographical zones of Europe according to the European Environment Agency (2000). The Alpine zone is divided between Alpine North (Scandes) and Alpine (the remaining parts distributed at lower latitudes, chiefly the Pyrenees, the Alps, the Carpathians and high-altitude areas in the Balkans). The final map layout was created in ArcGIS Desktop v. 10.7 (Esri, California, USA) and CorelDraw v. 20.1.0.707 (2018 Corel Corp.).


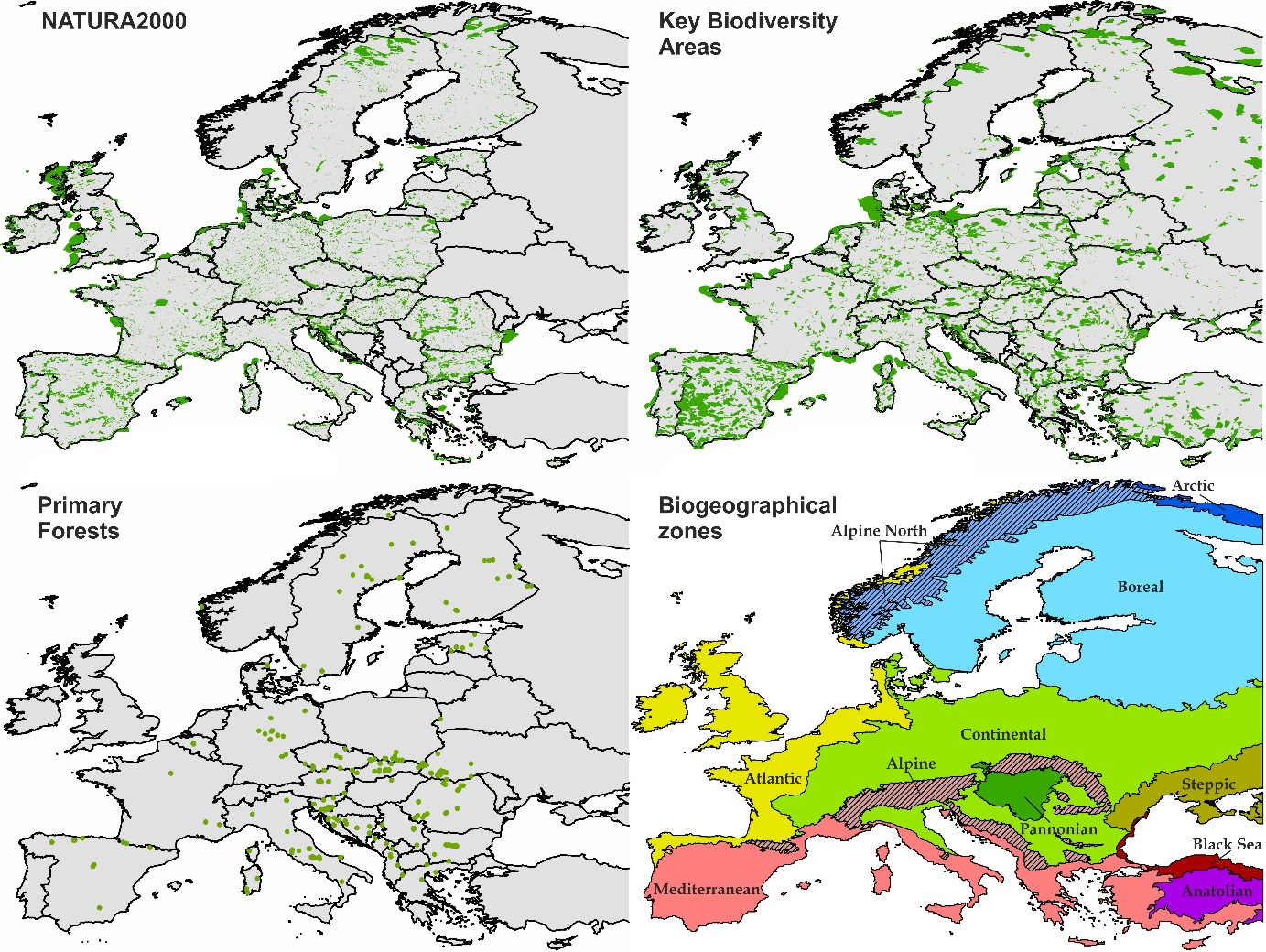


**Supplementary Figure S5**. Aggregate Climate Change (ACC) derived from changes in nine climate variables between the period 2081-2100 and 1961-1990. ACC % denotes relative aggregate climate change in each grid cell calculated as the fraction of maximum change defined as all variables reaching their continent-wide maximum. The residual ACC represent the residuals retained after the subtraction of the continent-wide spatial trend from original ACC values in each cell. Maps shown are based on the CLMcom-CLM4-8-17 RCM (see Table S1). The maps were created in R v. 4.0.4. (R Core Team, Vienna, Austria). The final layout was created in ArcGIS Desktop v. 10.7 (Esri, California, USA) and CorelDraw v. 20.1.0.707 (2018 Corel Corp.).

**
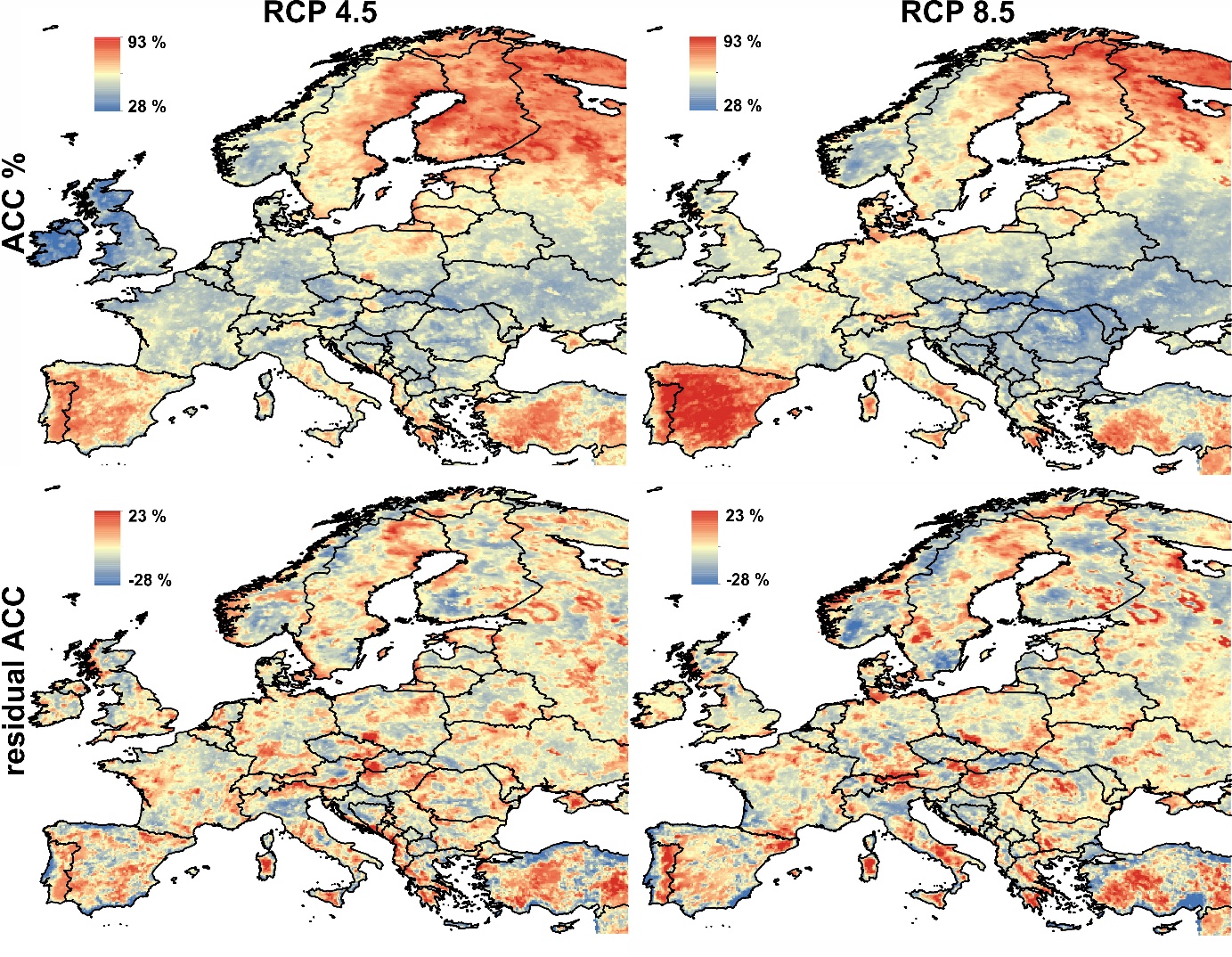
**

**Supplementary Figure S6.** Inter-model agreement of the location of different climate stability areas in Europe. Numbers denote the number of climate models which indicated the presence of climate significantly different from background. Data shown are for the RCP 4.5 and RCP 8.5, both for the period 2081-2100. The maps were created in R v. 4.0.4. (R Core Team, Vienna, Austria). The final layout was created in ArcGIS Desktop v. 10.7 (Esri, California, USA) and CorelDraw v. 20.1.0.707 (2018 Corel Corp.).


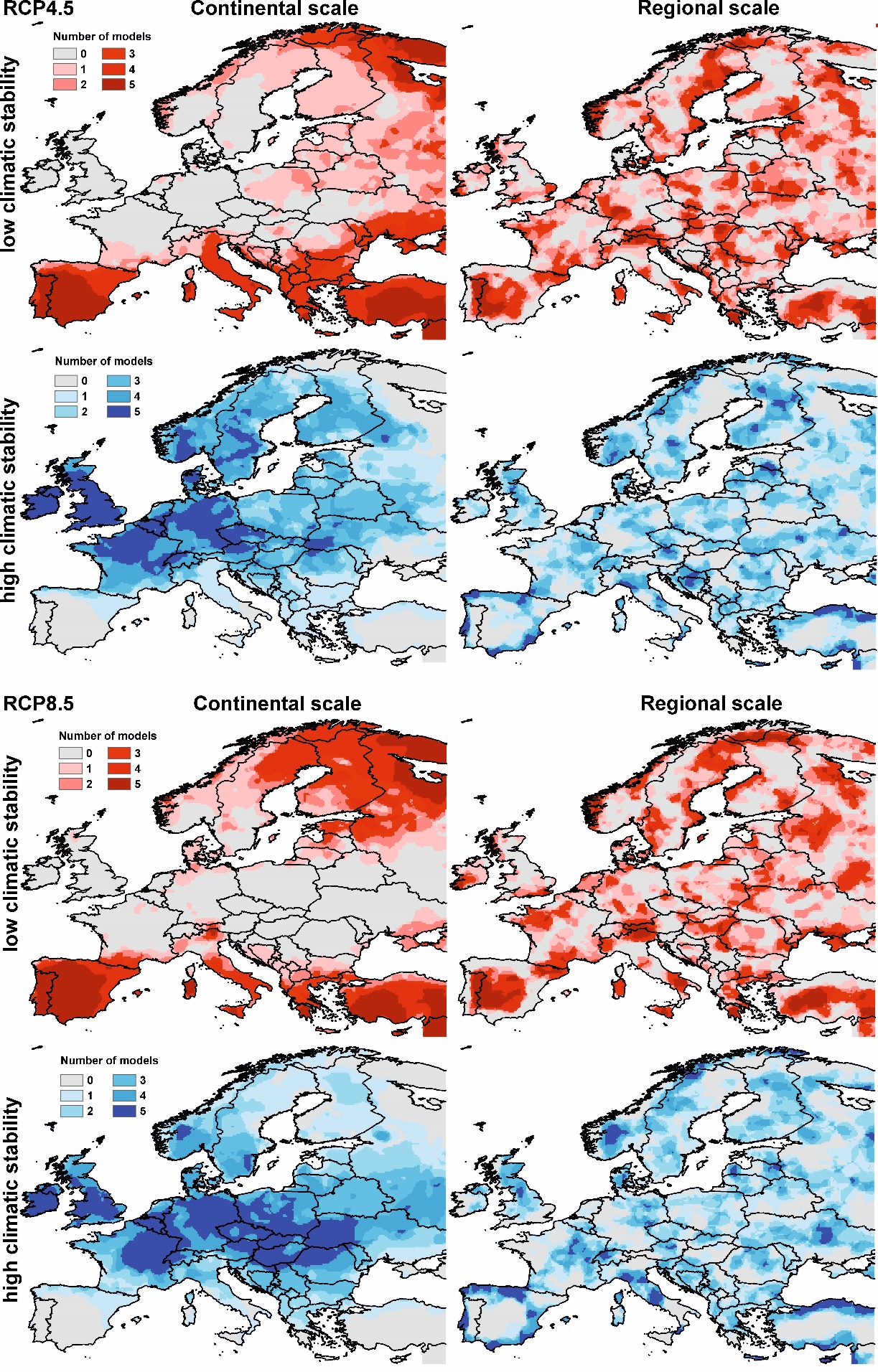


**Supplementary Figure S7.** Degree of robustness of locations with high and low climatic stability identified at regional and continental scales. Value range 0-30 indicates number of regional climate models, RCP scenarios and future time periods in which the feature was projected to occur in each grid cell. The total number of variants is given as: 5 RCMs × 2 RCPs × 3 time periods (2041-2061, 2061-2080, 2081-2100). The maps were created in R v. 4.0.4. (R Core Team, Vienna, Austria). The final layout was created in ArcGIS Desktop v. 10.7 (Esri, California, USA) and CorelDraw v. 20.1.0.707 (2018 Corel Corp.).

**
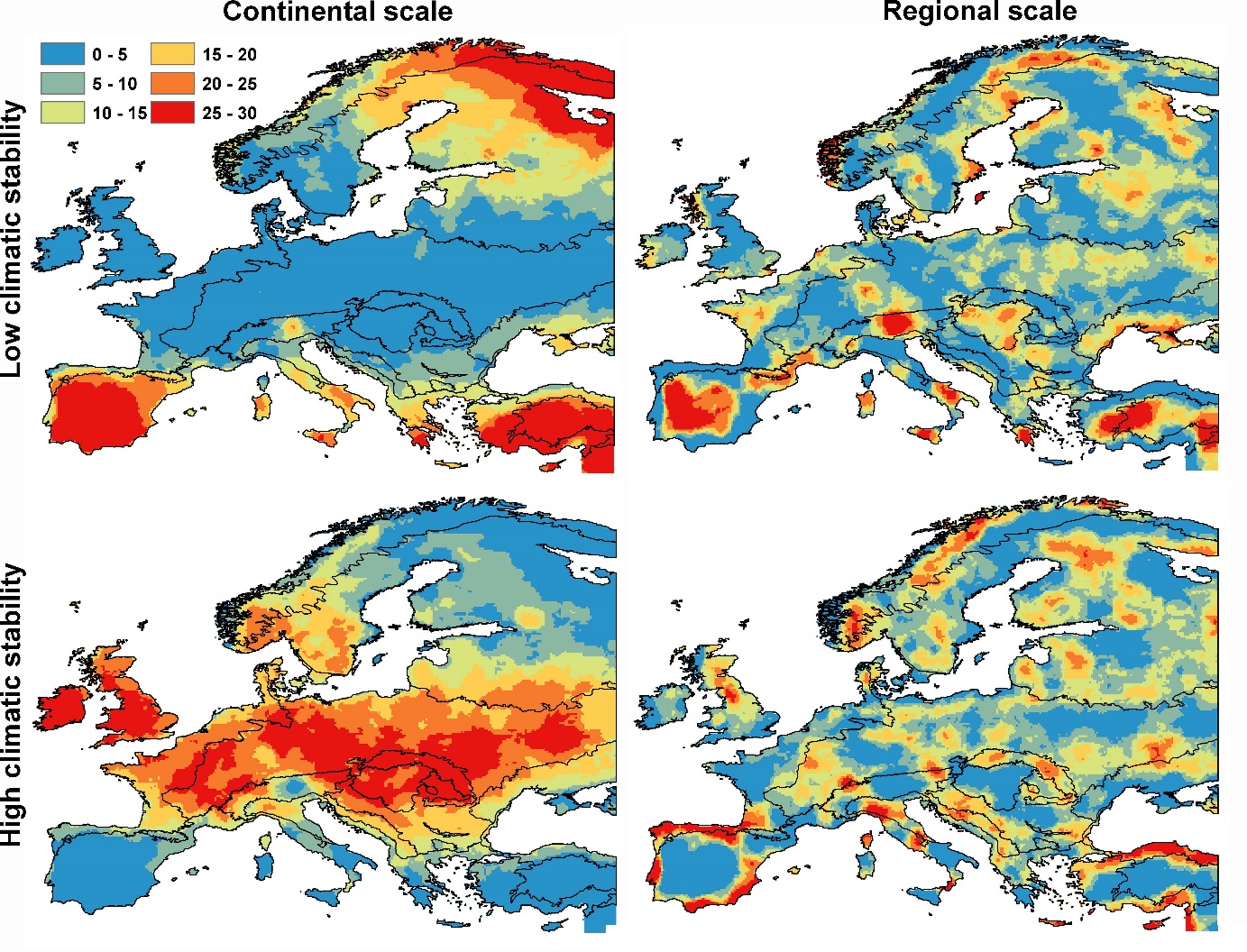
**

**Supplementary Figure S8.** Interaction of European global biodiversity hotspots with the continental-scale zones of low climatic stability. Indicated stability zones represent areas identified under a single (a) and both (b) RCPs used in this study as having climate with significantly low stability which persists throughout the 21^st^ century. Locations of the biodiversity sub-hotpots embedded within the Mediterranean Basin are indicated as well: BS – Baetic System, MLA – Maritime and Ligurian Alps, SCG – Central and Southern Greece, CR – Crete, ST – Southern Turkey, CY – Cyprus. The final layout was created in ArcGIS Desktop v. 10.7 (Esri, California, USA) and CorelDraw v. 20.1.0.707 (2018 Corel Corp.).

**
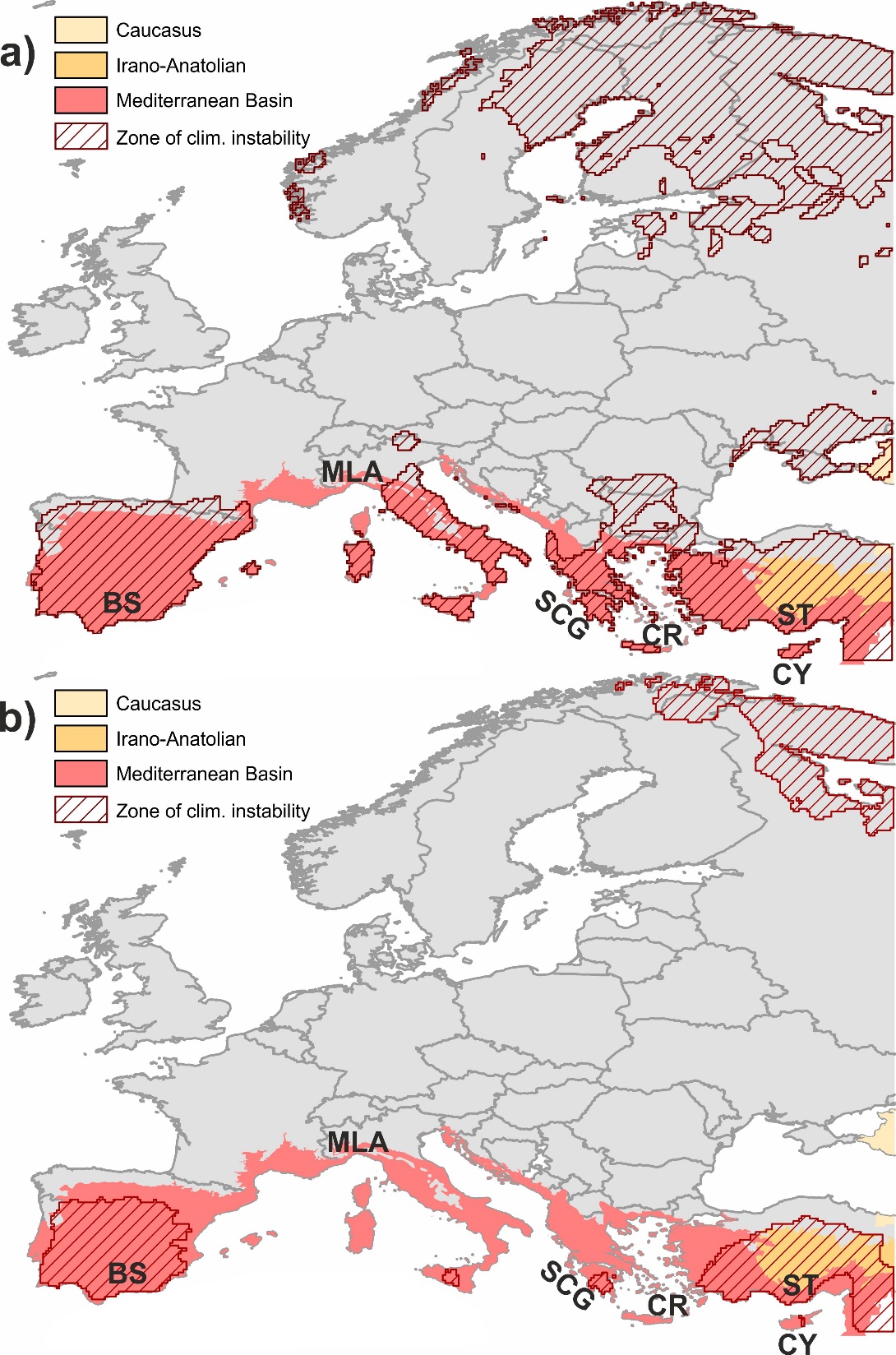
**

**Supplementary Figure S9.** The proportion of Natura 2000 (a), Key Biodiversity Areas (b) and European primary forests (c) present in areas with significantly low and high climatic stability at continental or regional scales, relative to background climate change. Climatic stability as predicted under RCP4.5 or RCP8.5 and persisting throughout 2041-2100. Horizontal dashed lines indicate European means for a given biodiversity feature. Arct – arctic, Bor – boreal, Alp N - Alpine north, Atl – Atlantic, Cont – continental, Pan – Pannonian, Alp – Alpine, Step – Steppic, Med – Mediterranean, Anat – Anatolian, Black – Black Sea


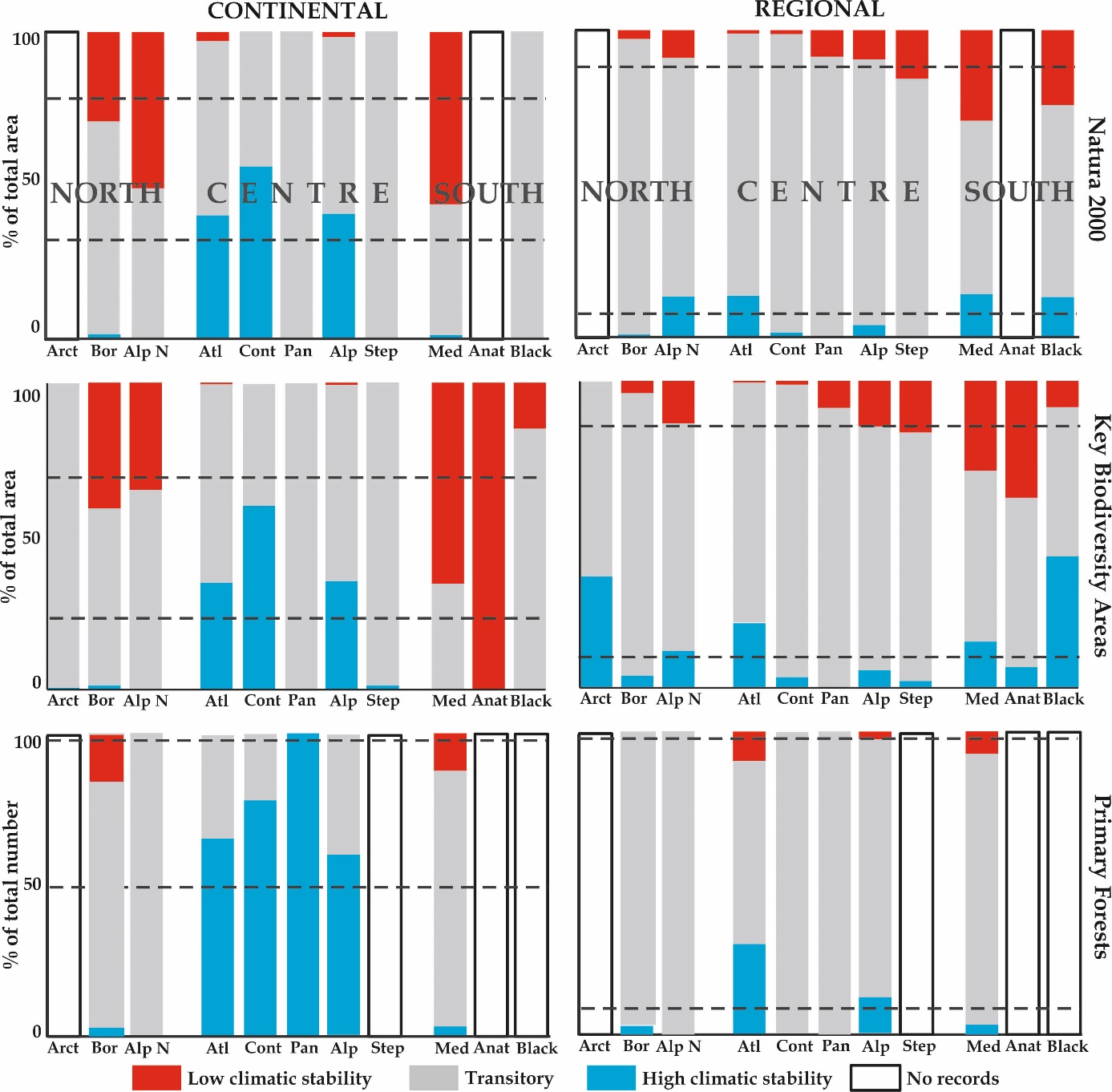


**Supplementary information B (code** **for the identification of areas of future climatic stability)**

**Example of a single climate model**

**Importing all climatic raster files for all target time periods:**

1961-1990 (Past climate - p)
2041-2060 (Future first period - f1)
2061-2080 (Future second period - f2)
2081-2100 (Future third period - f3).

In this example, we considered:

Nine climate variables (Table 1): Cont; DDa5; EQ; FFP; LLDP; MWMT; nDP; pr_ann; tasmax

One climate model: RCP 4.5 CNRM

Altogether, four time periods × nine climate variables produce 36 raster files

**library**(raster)

## Loading required package: sp

**library**(rasterVis)

## Loading required package: lattice

## Loading required package: latticeExtra

**library**(mgcv)

## Loading required package: nlme

##
## Attaching package: 'nlme'

## The following object is masked from 'package:raster':
##
## getData

## This is mgcv 1.8-31. For overview type 'help("mgcv-package")'.

dataroot = "C:/HotSpot_R/RCP45/DATA_CNRM"

p.list = **list.files**(dataroot, pattern="1961",full.names=T)
f1.list = **list.files**(dataroot, pattern="2041",full.names=T)
f2.list = **list.files**(dataroot, pattern="2061",full.names=T)
f3.list = **list.files**(dataroot, pattern="2081",full.names=T)

rp.list = **lapply**(p.list, raster)
rf1.list = **lapply**(f1.list, raster)
rf2.list = **lapply**(f2.list, raster)
rf3.list = **lapply**(f3.list, raster)


**for** (i **in** 1**:**9) {
 **NAvalue**(rp.list[[i]]) = -3.402823e+38
 **NAvalue**(rf1.list[[i]]) = -3.402823e+38
 **NAvalue**(rf2.list[[i]]) = -3.402823e+38
 **NAvalue**(rf3.list[[i]]) = -3.402823e+38
}

Example output for the variable Cont (climatic continentality) for four time periods:


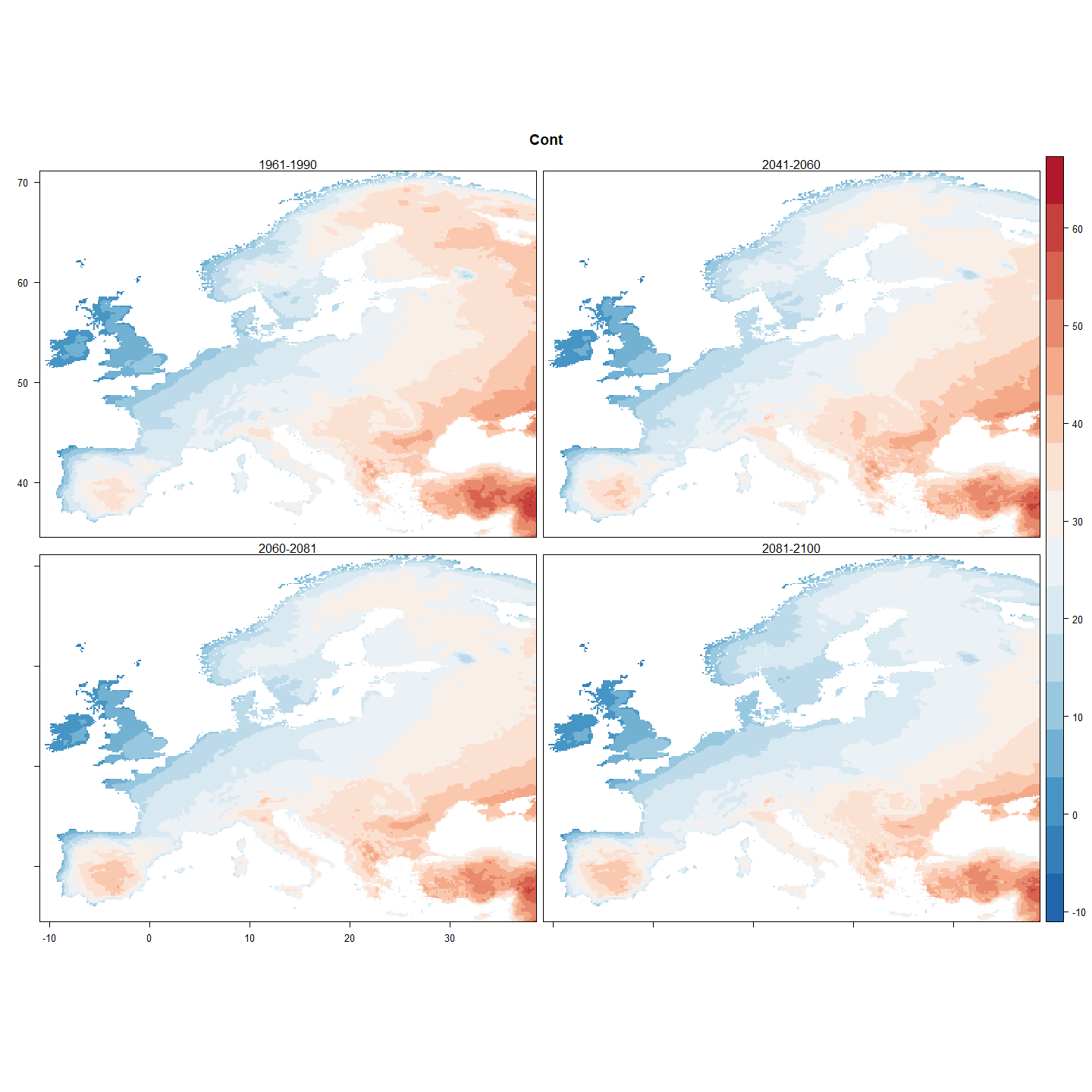


**Calculation of Standardize Euclidean Distance (SED):**

SED1_list = **list**()
SED2_list = **list**()
SED3_list = **list**()

**for** (i **in** 1**:**9){

 p = rp.list[[i]]
 f1 = rf1.list[[i]]
 f2 = rf2.list[[i]]
 f3 = rf3.list[[i]]

 minus1 = **abs**(f1 **-** p)
 minus2 = **abs**(f2 **-** p)
 minus3 = **abs**(f3 **-** p)

 q1 = **quantile**(minus1, probs = **c**(0.95))
 q2 = **quantile**(minus2, probs = **c**(0.95))
 q3 = **quantile**(minus3, probs = **c**(0.95))

 SED1 = (minus1 **/** q1)**^**2
 SED2 = (minus2 **/** q2)**^**2
 SED3 = (minus3 **/** q3)**^**2

 SED1[SED1**>**1] = 1
 SED2[SED2**>**1] = 1
 SED3[SED3**>**1] = 1

 SED1_list[[i]] = SED1
 SED2_list[[i]] = SED2
 SED3_list[[i]] = SED3
}

SED1r = **stack**(SED1_list)
SED2r = **stack**(SED2_list)
SED3r = **stack**(SED3_list)

Example output of SED for the three future time periods:


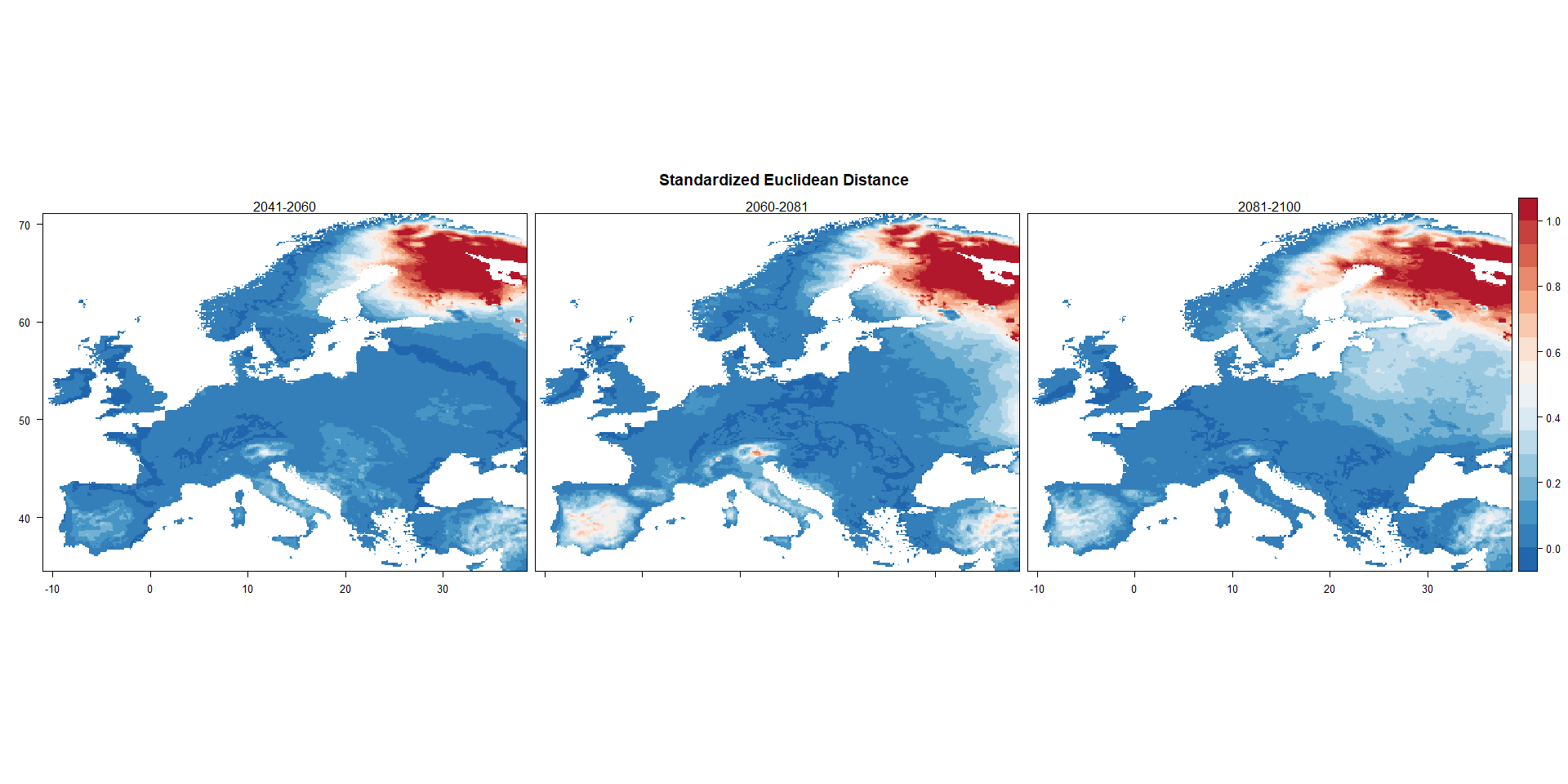


**Calculation of the Aggregate Climate Change**

CNRM_1 = **sqrt**(**calc**(**subset**(SED1r, 1**:**9), sum))
CNRM_2 = **sqrt**(**calc**(**subset**(SED2r, 1**:**9), sum))
CNRM_3 = **sqrt**(**calc**(**subset**(SED3r, 1**:**9), sum))

Visualization of Aggregate Climate Change for each time period and one climate model


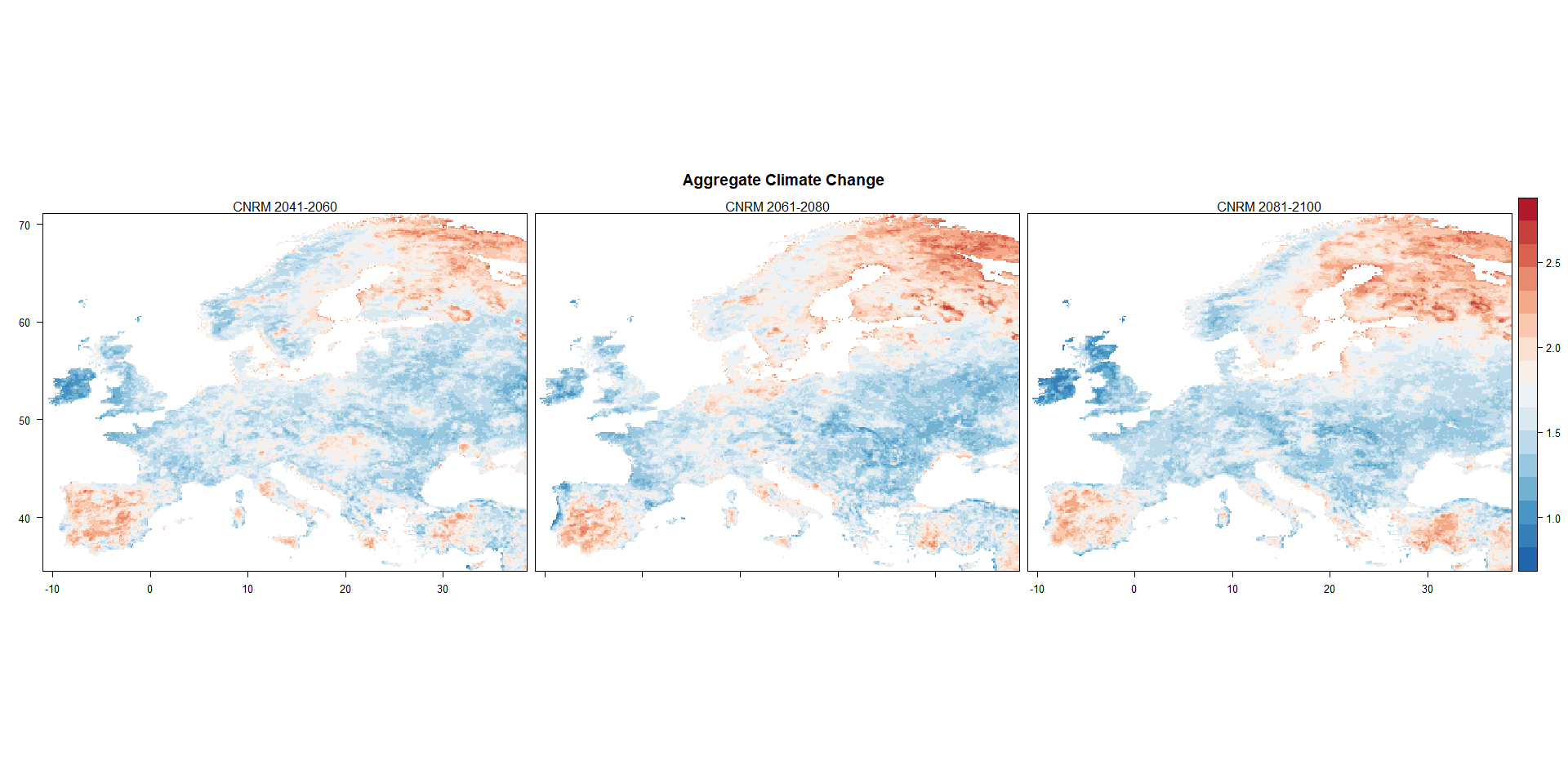


**Calculation of the relative Aggregate Climate Change (%)**

CNRM_1_per = (CNRM_1**/sqrt**(9))*****100
CNRM_2_per = (CNRM_2**/sqrt**(9))*****100
CNRM_3_per = (CNRM_3**/sqrt**(9))*****100

Visualization of relative Aggregate Climate Change (%) for three future time periods and one climate model:


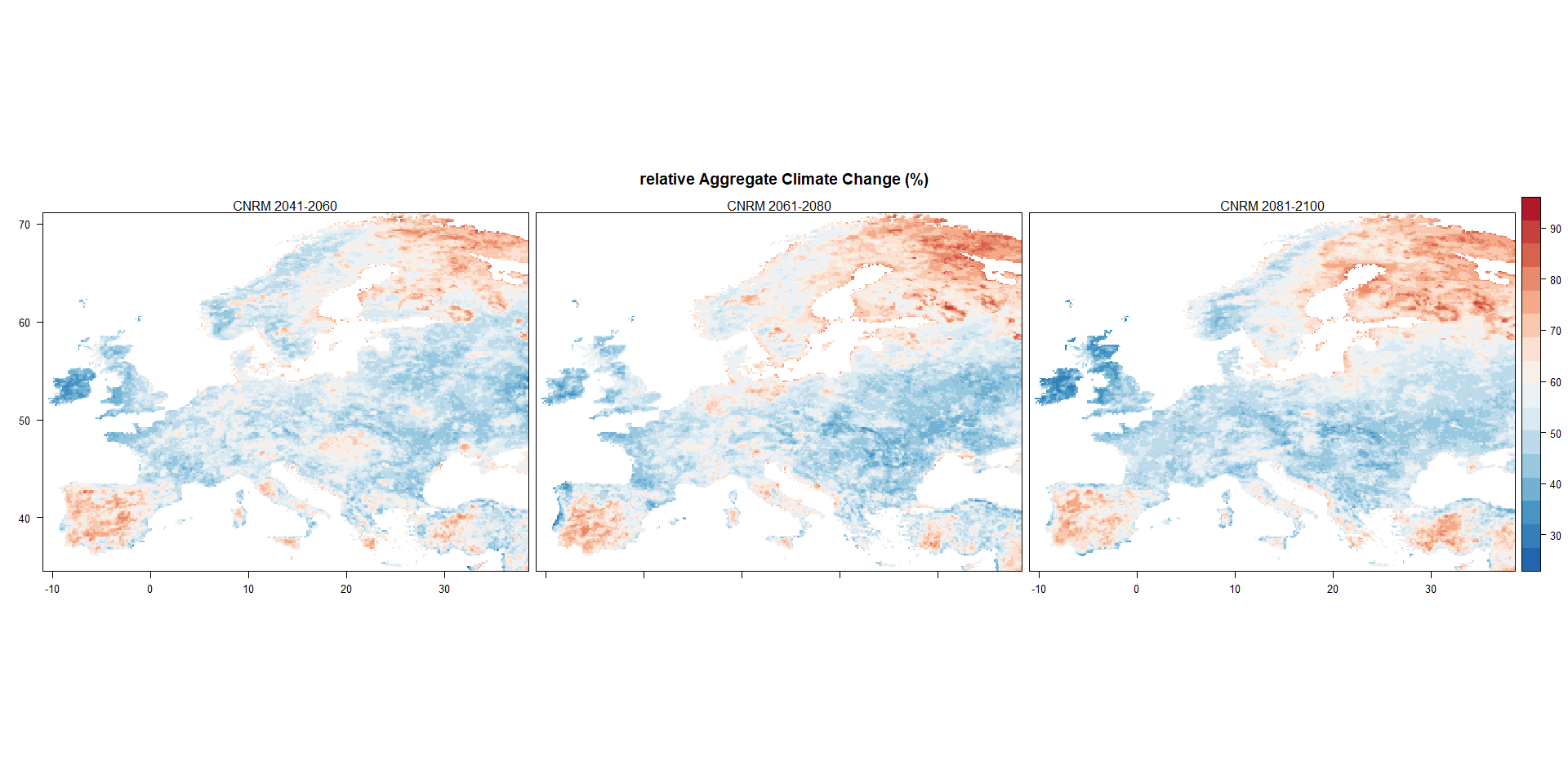


**Extracting the continental trend from ACC and calculating residual ACC**

CNRM_1_gam = **as.data.frame**(CNRM_1_per, xy = TRUE)
CNRM_2_gam = **as.data.frame**(CNRM_2_per, xy = TRUE)
CNRM_3_gam = **as.data.frame**(CNRM_3_per, xy = TRUE)

la = CNRM_1_gam**$**y
lo = CNRM_1_gam**$**x
ACC = CNRM_1_gam**$**layer
M1 = **gam**(ACC**~s**(la,lo, bs="sos"))
**sink**("CNRM_1_gam.txt")
**print**(**summary**(M1))

##
## Family: gaussian
## Link function: identity
##
## Formula:
## ACC ~ s(la, lo, bs = "sos")
##
## Parametric coefficients:
## Estimate Std. Error t value Pr(>|t|)
## (Intercept) 55.25078 0.01951 2832 <2e-16 ***
## ---
## Signif. codes: 0 '***' 0.001 '**' 0.01 '*' 0.05 '.' 0.1 ' ' 1
##
## Approximate significance of smooth terms:
## edf Ref.df F p-value
## s(la,lo) 48.92 49 2288 <2e-16 ***
## ---
## Signif. codes: 0 '***' 0.001 '**' 0.01 '*' 0.05 '.' 0.1 ' ' 1
##
## R-sq.(adj) = 0.579 Deviance explained = 57.9%
## GCV = 31.022 Scale est. = 31.003 n = 81455

**sink**()
CNRM_1_gam = **na.omit**(CNRM_1_gam)
CNRM_1_gam**$**residuals = **residuals**(M1)
CNRM_1_gam**$**fitted = **fitted**(M1)

la = CNRM_2_gam**$**y
lo = CNRM_2_gam**$**x
ACC = CNRM_2_gam**$**layer
M2 = **gam**(ACC**~s**(la,lo, bs="sos"))
**sink**("CNRM_2_gam.txt")
**print**(**summary**(M2))

##
## Family: gaussian
## Link function: identity
##
## Formula:
## ACC ~ s(la, lo, bs = "sos")
##
## Parametric coefficients:
## Estimate Std. Error t value Pr(>|t|)
## (Intercept) 56.45546 0.01872 3016 <2e-16 ***
## ---
## Signif. codes: 0 '***' 0.001 '**' 0.01 '*' 0.05 '.' 0.1 ' ' 1
##
## Approximate significance of smooth terms:
## edf Ref.df F p-value
## s(la,lo) 48.94 49 4159 <2e-16 ***
## ---
## Signif. codes: 0 '***' 0.001 '**' 0.01 '*' 0.05 '.' 0.1 ' ' 1
##
## R-sq.(adj) = 0.714 Deviance explained = 71.5%
## GCV = 28.554 Scale est. = 28.537 n = 81455

**sink**()
CNRM_2_gam = **na.omit**(CNRM_2_gam)
CNRM_2_gam**$**residuals = **residuals**(M2)
CNRM_2_gam**$**fitted = **fitted**(M2)

la = CNRM_3_gam**$**y
lo = CNRM_3_gam**$**x
ACC = CNRM_3_gam**$**layer
M3 = **gam**(ACC**~s**(la,lo, bs="sos"))
**sink**("CNRM_3_gam.txt")
**print**(**summary**(M3))

##
## Family: gaussian
## Link function: identity
##
## Formula:
## ACC ~ s(la, lo, bs = "sos")
##
## Parametric coefficients:
## Estimate Std. Error t value Pr(>|t|)
## (Intercept) 56.15081 0.01793 3131 <2e-16 ***
## ---
## Signif. codes: 0 '***' 0.001 '**' 0.01 '*' 0.05 '.' 0.1 ' ' 1
##
## Approximate significance of smooth terms:
## edf Ref.df F p-value
## s(la,lo) 48.91 49 5068 <2e-16 ***
## ---
## Signif. codes: 0 '***' 0.001 '**' 0.01 '*' 0.05 '.' 0.1 ' ' 1
##
## R-sq.(adj) = 0.753 Deviance explained = 75.3%
## GCV = 26.21 Scale est. = 26.193 n = 81455

**sink**()
CNRM_3_gam = **na.omit**(CNRM_3_gam)
CNRM_3_gam**$**residuals = **residuals**(M3)
CNRM_3_gam**$**fitted = **fitted**(M3)

CNRM_1_gam_brick = **rasterFromXYZ**(CNRM_1_gam)
CNRM_1_res = **raster**(CNRM_1_gam_brick, layer=2)
CNRM_1_fit = **raster**(CNRM_1_gam_brick, layer=3)

CNRM_2_gam_brick = **rasterFromXYZ**(CNRM_2_gam)
CNRM_2_res = **raster**(CNRM_2_gam_brick, layer=2)
CNRM_2_fit = **raster**(CNRM_2_gam_brick, layer=3)

CNRM_3_gam_brick = **rasterFromXYZ**(CNRM_3_gam)
CNRM_3_res = **raster**(CNRM_3_gam_brick, layer=2)
CNRM_3_fit = **raster**(CNRM_3_gam_brick, layer=3)

Visualization of the residual Aggregate Climate Change


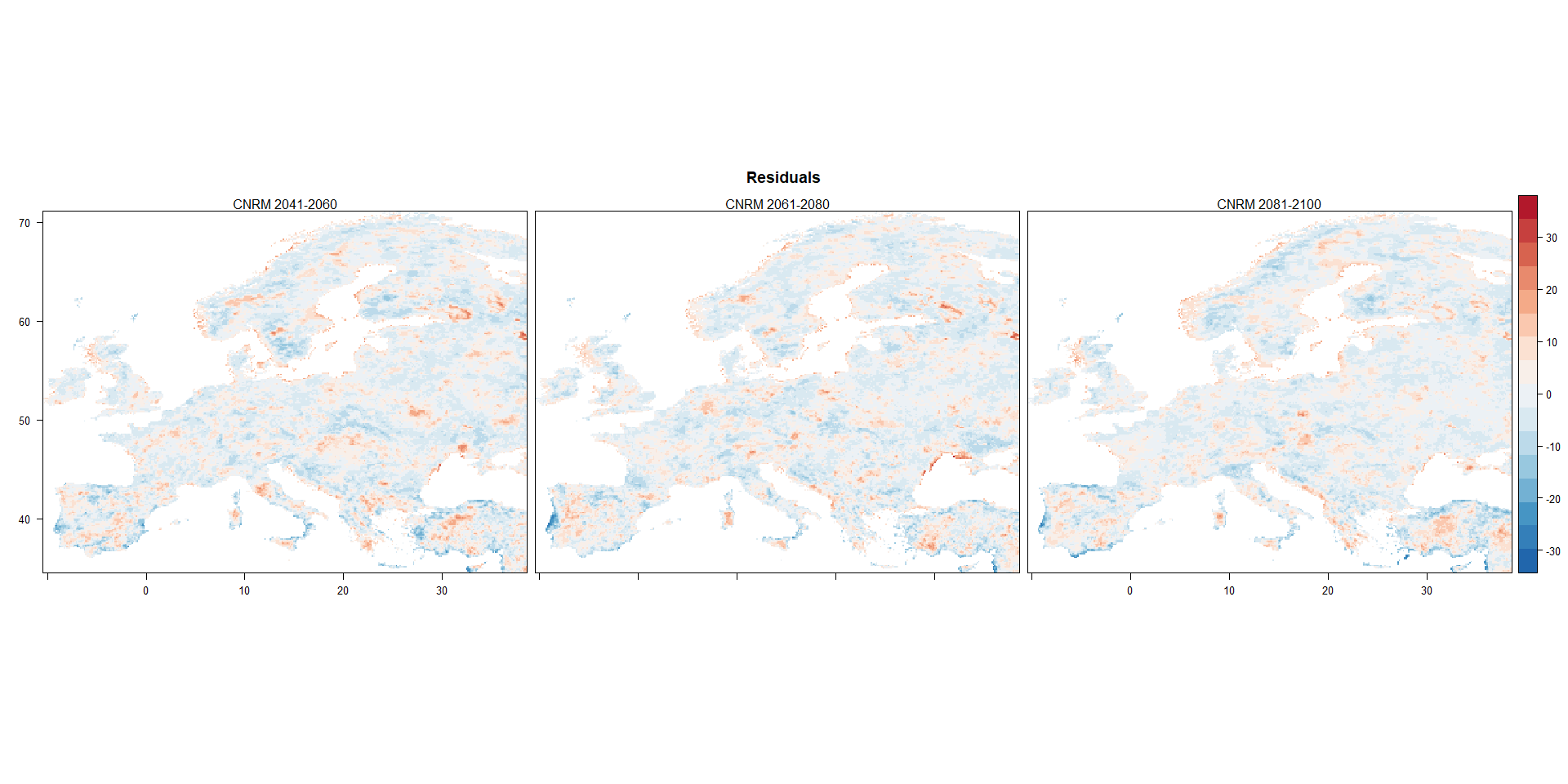


The remaining analysis, i.e., detection of zones of significantly high and low climatic stability using the Gettis-Ord statistics, was conducted in ArcGIS Desktop v. 10.7 (Esri, California, USA)
